# An insight into carcinogenic activity and molecular mechanisms of Bis(2-ethylhexyl) phthalate

**DOI:** 10.3389/ftox.2024.1389160

**Published:** 2024-07-23

**Authors:** Gelsomina Pillo, Federico Aldrovandi, Ada Mescoli, Giangabriele Maffei, Maria Grazia Mascolo, Monica Vaccari, Annamaria Colacci

**Affiliations:** ^1^ Agency for Prevention, Environment and Energy (Arpae), Bologna, Italy; ^2^ Department of Medical and Surgical Sciences, Alma Mater Studiorum, University of Bologna, Bologna, Italy; ^3^ Alma Mater Institute on Healthy Planet, University of Bologna, Bologna, Italy; ^4^ Department of Biological, Geological and Environmental Sciences, University of Bologna, Bologna, Italy

**Keywords:** bis(2-ethylhexyl) phthalate, non-genotoxic carcinogens, alternative methods, transcriptomics, toxicogenomics, cell transformation assay, cytotoxicity, transformics

## Abstract

Di(ethylhexyl) phthalate (DEHP) is a ubiquitous environmental contaminant to which humans are exposed via multiple routes. Human health risk assessments for this substance have recently been updated, focusing on reproductive toxicity, including DEHP, in the list of chemicals classified as carcinogenic, mutagenic, or toxic to reproduction (CMR). Moreover, DEHP has also been defined as probably and possibly carcinogenic to humans based on its carcinogenicity in rodents. However, the mechanism of action of DEHP and its relevance in humans remain unclear. Rodent data suggests that DEHP induces cancer through non-genotoxic mechanisms related to multiple molecular signals, including PPARα activation, perturbation of fatty acid metabolism, induction of cell proliferation, decreased apoptosis, production of reactive oxygen species, and oxidative stress. According to the DEHP toxicological dataset, several *in vitro* cell transformation assays have been performed using different protocols and cellular models to produce different results. This study aimed to evaluate the carcinogenic potential of DEHP by using the A31-1-1 BALB/c-3T3 cell line in a standard cell transformation assay. Additionally, transcriptomic analysis was performed to explore the molecular responses and identify the affected toxicological pathways. Although DEHP treatment did not induce transformation in BALB/c-3T3 cells, the transcriptomic results revealed significant modulation of several pathways associated with DEHP metabolism, tissue-specific functions related to systemic metabolism, and basal cellular signaling with pleiotropic outcomes. Among these signaling pathways, modulation of cell-regulating signaling pathways, such as Notch, Wnt, and TGF-β, can be highlighted. More specific modulation of such genes and pathways with double functions in metabolism and neurophysiology underlies the well-known crosstalk that may be crucial for the mechanism of action of DEHP. Our findings offer evidence to support the notion that these models are effective in minimizing the use of animal testing for toxicity assessment.

## 1 Introduction

Di(2-ethylhexyl) phthalate (DEHP; CAS No. 117-81-7), a chemical belonging to the phthalate family, is a synthetic substance that is commonly incorporated into plastics to increase their flexibility. DEHP is particularly noteworthy as it is the index compound of the class for group-tolerable daily intake (TDI) calculations because it possesses the most extensive toxicological dataset among its counterparts.

Phthalates are widely used in various commercial products and as packaging materials. Because they are non-covalently bonded to polyvinyl chloride (PVC), they can be easily released by plastics in the surrounding matrices, generating widespread pollution that affects the environment worldwide and poses a greater exposure risk to the general population (Rowdhwal and Chen, 2018). DEHP metabolites have been detected in human bodily fluids (Wang et al., 2019). DEHP can be absorbed via the dermal, inhalation, and oral routes. Once ingested, DEHP is rapidly metabolized in the liver, producing approximately 30 different metabolites that are promptly excreted in the urine as glucuronide conjugates (Hauser and Calafat, 2005). DEHP is first hydrolyzed to mono(2-ethylhexyl) phthalate (MEHP). Subsequently, MEHP is metabolized by cytochrome P450 enzymes, specifically human CYP2C9(*)1, CYP2C9(*)2, CYP2C19, and rat CYP2C6 (Choi et al., 2012) to generate oxidative and dealkylated metabolites. The most common metabolites of MEHP are mono(2-ethyl-5-hydroxyhexyl) phthalate (MEHHP), mono(2-ethyl-5-oxohexyl) phthalate (MEOHP), and mono(2-ethyl-5-carboxypentyl) phthalate (MECPP). These metabolites have been frequently detected in biological samples (Koch et al., 2006).

Over time, research has hinted at the potential toxicological and carcinogenic effects of phthalates in humans, prompting regulatory measures in the European Union to limit their use. However, the evidence remains suggestive rather than conclusive. The carcinogenic potential of DEHP has been assessed by various regulatory authorities, and conclusions have changed over time.

DEHP causes cancer and reproductive, developmental, nerve, immune, and endocrine disruptions in rodents (Rowdhwal and Chen, 2018). After much debate, 11 types of phthalates, including BBP, DBP, and DEHP, have been classified as reproductive toxicants in category 1 B (suspected reproductive toxicants) according to the carcinogenic, mutagenic, or toxic to reproduction (CMR) classification (SCHEER, 2019).

The overall weight of evidence suggests that DEHP is not genotoxic, but can induce hepatic tumors in mice and rats, with some inconclusive evidence of testicular and pancreatic tumors (Madia et al., 2020; NTP, 2021) (Table 1). However, extrapolation of these results to humans has not yet been proven.

**TABLE 1:** Comprehensive genotoxicity and carcinogenicity assessment results of DEHP from the EURL ECVAM genotoxicity and carcinogenicity consolidated database of Ames-negative chemicals (Koch et al., 2006).

Genotoxicity and carcinogenicity assay	Overall result^a^
**AMES Tests** (OECD 471 TG): both in the presence and absence of an exogenous source of metabolic activation	Negative
***In vitro* Mammalian Cell Gene Mutation (MCGM) assays:** mouse lymphoma Tk+/− mutation assay, Hprt mutation assay and human TK6 cells mutation assay	Negative
***In vitro* Mammalian Cell Micronucleus Test**	Negative
***In vitro* Comet Assay**	Negative
***In vivo* Mammalian Cell Micronucleus Test**	Negative
***In vivo* Comet Assay**	Negative
**Transgenic rodent gene mutation assays (TGR)**	Equivocal
***In vivo*** **unscheduled DNA synthesis**	Negative
***In vivo*** C**omet Assay:** in stomach, colon, liver, kidney, bladder, lung, brain and bone marrow of male mice via gavage; in stomach, liver and bone marrow of male rats via gavage	Negative
**Rodent Carcinogenicity**	Positive

^a^ Overall result refers to the final call provided by EURL ECVAM applying specific criteria, including quality and the robustness of the study.

The main mechanism involved in rodent hepatotoxicity and hepatocarcinogencity of DEHP is transactivation of peroxisome proliferator-activated receptor alpha (PPARα) signaling, which is physiologically involved in the regulation of lipid metabolism and glucose homeostasis. Perturbation of this signaling pathway is thought to have little or no relevance in humans (Hasmall et al., 2000; Isenberg et al., 2000; Colacci et al., 2023).

The current body of evidence does not conclusively establish a causal relationship between DEHP exposure and cancer development. Although many scientists acknowledge that the lack of carcinogenicity of DEHP in humans is primarily based on indirect evidence and peroxisome proliferation cannot be definitively identified as the sole mechanism of DEHP carcinogenicity, the possibility of DEHP tumorigenesis via non-PPARα pathways, such as nuclear factor kappa B (NFκB), androstane receptor (CAR), and pregnant X receptor (PXR), remains unclear. *In vivo* studies employing PPARα-null mice and PPARα-humanized mouse carcinogenicity tests have yielded conflicting results, with some evidence of DEHP hepatocarcinogenesis in both genotypes; however, these findings remain controversial (Ito et al., 2007; Corton et al., 2018; Foreman et al., 2021a; Foreman et al., 2021b; Colacci et al., 2023).

Additionally, the tumor-promoting activity of DEHP has been investigated, and research points to its potential to promote the progression of hormone-related lesions and increase the risk of various cancers, including breast (Wu et al., 2021; Mukherjee Das et al., 2022), thyroid (Marotta et al., 2019; Liu et al., 2020), ovarian (Leng et al., 2021), and prostate (Chuang et al., 2020; Colacci et al., 2023; Guo et al., 2023) cancers.

Based on these results, the International Agency for International Research on Cancer (IARC) and US-EPA classified DEHP as a possible carcinogen (2 B substance suspected of causing cancer), subject to multiple mechanisms and pathways simultaneously involved, related to a non-genotoxic Mode of Action (MoA) (IARC, 2013). However, the European Chemicals Agency (ECHA) does not warrant classification for carcinogenicity, as the risk assessment conducted under Regulation (EC) N° 1907-2006 (REACH) does not consider these data owing to the derivation of the Dose-Response for Exposure Assessment (DNELs) for DEHP from reproductive toxicity data.

In addition to *in vivo* carcinogenicity data, controversial results were obtained by testing DEHP in cell transformation assays (CTAs) (Supplementary Table S1). CTA is a valuable *in vitro* test used to assess the carcinogenic potential of both genotoxic and non-genotoxic chemicals as well as environmental agents. CTAs use cultured mammalian cells to measure their ability to undergo malignant transformation in response to a test substance (Colacci et al., 2023). All CTAs provide an easily detectable endpoint for morphological transformation, anchoring chemical exposure to the acquisition of the malignant phenotype. Moreover, the application of transcriptomic approaches to CTAs offers a powerful means to elucidate the mechanisms underlying the carcinogenic potential of the tested substances (Mascolo et al., 2018; Pillo et al., 2022; Colacci et al., 2023).

Although CTA is considered insufficient for classifying chemicals as carcinogens on its own, it is a crucial component integrated approach to testing and assessment (IATA) for non-genotoxic carcinogens (NGTxC) based on leveraging omics technology, particularly transcriptomics, to gain a more nuanced mechanistic understanding of the behavior exhibited by the tested chemical (Jacobs et al., 2016; Jacobs et al., 2020; Oku et al., 2022). There are currently three widely used *in vitro* models for testing chemically induced transformations, which have been considered for inclusion in the IATA for NGTxC: the SHE model, BALB/c 3T3 model, and Bhas42 CTA, differing in the degree of cell progression towards transformation.

There is still an ongoing debate on whether the three CTA models are interchangeable or whether there should be criteria guiding the choice of one over the other based on their peculiarities and the characteristics of the tested chemicals (Colacci et al., 2023), as there are still some critical issues related to the use of the current experimental protocols of CTA.

In the absence of approved test guidelines, the OECD issued two guidance documents endorsing the use of CTA based on SHE and Bhas 42 cells. Additionally, ECVAM recommended a protocol for BALB/c 3T3 CTA following a pre-validation study (Tanaka et al., 2012), aiming to encourage feedback from further studies exploring the transforming abilities of chemicals to enhance the experimental protocols (Colacci et al., 2023).

In this context, DEHP is a paradigmatic compound that can be used to address critical issues.

DEHP has been listed as a potential non-genotoxic carcinogen and has been tested in rodents and two CTA models, yielding varied and inconclusive results, demonstrating primarily positive results in the SHE CTA but producing negative outcomes in the BALB/c 3T3 CTA (Colacci et al., 2023). The mechanisms underlying DEHP toxicity, including the initiating molecular event and the type of receptor involved, have not yet been fully elucidated. Furthermore, DEHP is a prototype chemical compound whose low solubility may lead to procedural issues in *in vitro* tests in cell cultures, according to good *in vitro* practices for the development and implementation of *in vitro* methods for regulatory use in human safety assessments (OECD, 2018).

Therefore, to enhance the full utilization of CTA in IATA for NGTxC, we conducted a study on DEHP to understand the reasons for the discrepancies observed in various CTA tests and to identify its mechanism of action as a possible non-genotoxic carcinogen.

To achieve our objective, a standard CTA protocol using A-31-1-1 BALB/c-3T3 cells (Sasaki et al., 2012a; Corvi et al., 2012; Tanaka et al., 2012) was conducted, followed by transcriptomic analysis using the so-called transformics assay.

Transformics provides a comprehensive view of the entire process from chemical exposure to the final outcome, thereby elucidating the molecular mechanisms underlying oncotransformation. Gene modulation data were collected at key time points throughout the experimental protocol, allowing for detailed analysis of the molecular events driving the transformation process.

The transformics approach was developed to bridge gaps in mechanistic knowledge related to *in vitro* cell transformation, reconciling apparently conflicting data from CTA studies, supporting the integration of CTA within the IATA for NGTxC, and serving as a foundation for refining thresholds derived from *in vitro* experiments.

Indeed, the application of transcriptomic analysis to CTA has highlighted a cascade of key molecular events underlying *in vitro* oncotransformation, mirroring critical steps observed in human cancer progression. This comprehensive understanding, extensively discussed previously (Colacci et al., 2023), underscores the relevance of CTA results in human cancer pathogenesis and affirms the translational potential of these findings (Colacci et al., 2023).

Furthermore, transcriptomic analysis applied to CTA revealed the activation of receptor-mediated pathways crucial for metabolic processes, facilitating both bioactivation and detoxification of chemicals. This approach also provides insights into the molecular initiating events that drive chemically induced toxicity.

This investigation was intended to provide essential information for evaluating the feasibility of the proposed method for fulfilling the criteria for regulatory toxicology. These results are critical for endorsing the potential incorporation of this method into an integrated approach to testing and assessment (IATA) designed for NGTxC (Jacobs et al., 2020; Oku et al., 2022; Pillo et al., 2022). In addition, this study aimed to elucidate the toxicological behavior of DEHP.

## 2 Materials and methods

### 2.1 Cells

Mouse embryo BALB/c 3T3 fibroblasts (clone A31-1-1) were obtained from the Health Science Research Resource Bank and were stored in liquid nitrogen. Cells at passage three were used for the preliminary cytotoxicity assay, whereas cells at passage one were used for CTA. Cells were seeded at a density of 125,000 cells/T75 flasks. Cells were cultured until they reached 70% confluence in M10F medium, which consisted of Minimum Essential Medium (MEM) supplemented with 10% Fetal Bovine Serum (FBS; Gibco BRL) and 1% 10,000 U/mL penicillin–10 mg/mL streptomycin.

### 2.2 Chemicals

Bis(2-ethylhexyl) phthalate (PESTANAL^®^), an analytical standard (DEHP, CAS No: 117-81-7, ≥98.0% purity, SIAL 36735), was used. Dimethyl Sulfoxide (DMSO, CAS number 67-68-5, Hybri-max Sterile, Sigma/D2650) was employed as the vehicle and solvent for the tested chemicals. Several studies have identified challenges in conducting assays for DEHP, particularly related to its poor miscibility and solubility in polar solvents despite the use of organic solvents as vehicles. In our literature review, we observed that several DEHP CTAs were performed at high concentrations, and many studies used 0.1% DMSO or other solvents as vehicles (Supplementary Table S1). Therefore, in this study, particular attention was paid to the dissolution of DEHP in cell media, leading to the use of a final concentration of 0.5% DMSO.

A concentrated solution of the chemicals in DMSO was prepared and serial dilutions were prepared from this solution. Vigorous vortexing was performed for approximately 5 min to ensure complete solubilization of the test items. During this experiment, DEHP was readily dissolved in DMSO without any increase in the turbidity.

The solubility of DEHP in water is 0.00003% (23.8°C); therefore, its solubility might decrease as the volume of DMSO decreases and the volume of cell culture medium increases.

The dissolution behavior of DEHP in DMSO and the stability of the stock solutions in cell medium were evaluated using a simple test and direct visual observation. The working solutions were incubated under test conditions (37°C, 5% CO2, and 90% relative humidity) for 72 h, and periodic checks were conducted to detect the presence of precipitates.

The working solutions were obtained by two groups of dilutions of the DMSO stock solutions in M10F: 1:1,000 and 1:200, resulting in final DEHP concentrations of 100, 75, and 25 μg/mL for each group. At the end of the procedure, solutions with a final DMSO concentration of 0.1% exhibited turbidity, and small oil droplets formed in the suspension were faintly visible to the naked eye.

Therefore, the final DMSO concentration of 0.5% was deemed more suitable for this experiment.

### 2.3 Transformics experimental protocol

The experimental protocol included a preliminary cytotoxicity assay, cell transformation assay including a concurrent cytotoxicity test, and transcriptomic experiment.

#### 2.3.1 Preliminary cytotoxicity assays

Two preliminary cytotoxicity assays were performed, covering a concentration range of 0.05–100 μg/mL, corresponding to 0.05 μL/mL to 102 μL/mL, in order to identify the range of DEHP concentrations to be tested in further experiments. Based on the results of the preliminary cytotoxicity assay, the following concentrations were used in the cell transformation assay: 2.79 μg/mL, 6.99 μg/mL, 17.48 μg/mL, 22.73 μg/mL, and 29.55 μg/mL. Transcriptomic experiments were conducted using cells treated with a cytotoxic concentration of DEHP 19.7 μg/mL for 24 h and 72 h.

#### 2.3.2 Cell transformation assay

The transformation assay was performed by applying the standard BALB/c-3T3 A-31-1-1 CTA ECVAM DB-ALM Protocol N. 137 (Sasaki et al., 2021a; Corvi et al., 2012; Tanaka et al., 2012; IARC, 2013; Mascolo et al., 2018). Cells in the logarithmic growth phase were seeded at a density of 1 × 10^^4^ cells/60 mm dish, with 10 dishes per treatment, in M10F culture media. After 24 h, the cells were exposed to the test compounds for 72 h. Untreated and solvent-treated cells served as negative controls, whereas MCA-treated cells were used as positive controls.

From day 8 post-seeding, the culture medium was replaced twice a week with DF2I2F, containing a low concentration of FBS (2%) and insulin (final concentration, 2 μg/mL). After 24 days post-seeding, no further medium changes were undertaken on day 31-32 post-seeding, and the plates were fixed with methanol and stained with 0.04% Giemsa stain. The occurrence of transformed Type III foci, characterized by deep basophilic staining, random cell orientation, dense multilayering of cells, and invasion into the surrounding contact-inhibited monolayer, was assessed using an optical microscope (Sasaki et al., 2012b).

#### 2.3.3 Transcriptomics experiment

##### 2.3.3.1 Total RNA extraction

Cells in the logarithmic phase of growth were seeded at a density of 1 × 10^4^ cells per 60 mm diameter dish using the CTA culture protocol. Twenty-four hours after seeding, cells were treated with 19.70 μg/mL DEHP or 0.5% DMSO as a control. Total RNA was isolated after 24 h and 72 h of exposure using TRIzol Reagent (Invitrogen, San Diego, CA, United States), followed by purification with an RNeasy affinity column (Qiagen, Valencia, CA, United States) according to the manufacturer’s instructions. RNA quality was assessed using an Agilent 4200 TapeStation system (Agilent RNA ScreenTape Analysis Kit) and NanoDrop OneC. Four type 1 biological replicates were obtained for each treatment (19.70 μg/mL DEHP and 0.5% DMSO).

##### 2.3.3.2 Total RNA labeling and hybridization

cRNA was labeled, purified, and hybridized on oligonucleotide slides (SurePrint G3 Mouse Gene Expression v2 8 × 60 K Microarray Kit) using the Low Input Quick Amp Labeling Kit, version 6.9.1, December 2015 (Agilent Technologies, Santa Clara, CA, United States) (www.genomics.agilent.com HYPERLINK http://www.chem.agilent.com/, accessed on 13 Oct 2023). Four arrays were hybridized with the treated cell lysate, and four with the control lysate, for each time points. Slides were scanned using an Agilent SureScan Microarray Scanner G2600D.

##### 2.3.3.3 Statistical analysis of microarray data

The image data were extracted using Feature Extraction Project software and analyzed using Agilent GeneSpring 14.9.1. For this study, differentially expressed genes were identified according to the following criteria: unpaired t-test p (Corr) cut-off = 0.05, with Benjamini Hochberg False Discovery Rate correction. In addition, a t-test unpaired p (Corr) cut-off = 0.05 adjusted by Bonferroni was also performed in order to make a comparison.

##### 2.3.3.4 Tools of biological interpretation

The lists of differentially expressed genes were imported into MetaCore software V6.34 (Clarivate Analytics (https://portal.genego.com/, accessed on 15 Oct 2023). Enrichment analysis was performed using the Analyze Single Experiment workflow with a fold-change cutoff of 1.5.

### 2.4 Immunofluorescence staining

2.5 × 10^4^ BALB/c 3T3 fibroblasts, clone A31-1-1 were cultured in ibidi µ-Slide 8 Wells, fixed in 4% paraformaldehyde for 30 min and then permeabilized with 0.2% Triton X-100. The cells were then treated with blocking solution (dPBS + 2.5% BSA) for 20 min at room temperature. The cells were then incubated with the primary antibodies (ab61182; Abcam, Shanghai, China) at room temperature for 1 h. The cells were then incubated with a secondary fluorescent-conjugated IgG (Alexa Fluor 488- IgG) (ab150077, Abcam) at room temperature for 1 h. The primary antibody dilution was 1:200, and the secondary antibody dilution was 1:500. After 1 h, the cells were washed thrice with dPBS. Hoechst staining was used to counterstain the nuclei. An inverted fluorescence microscope was used to capture the images.

## 3 Results

### 3.1 Cytotoxicity assay

**FIGURE 1:**
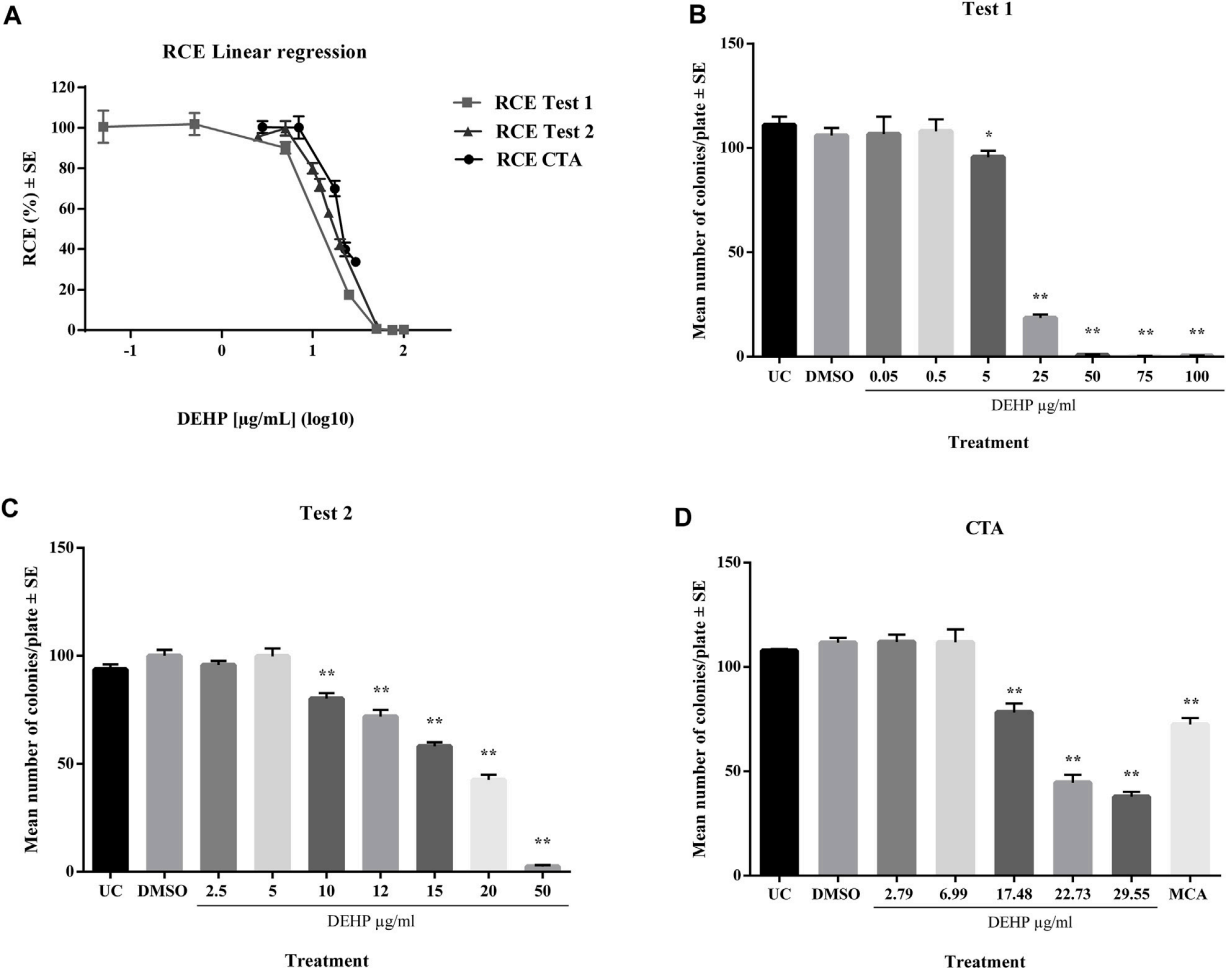
**(A)** Comparison of cytotoxicity assay results expressed as RCE linear regression (χ2-test). **(B)** Preliminary cytotoxicity assay 1 results expressed as the mean number of colonies ±SE. *p ≤ 0.05 vs. vehicle control, t-test. **p ≤ 0.01 vs. vehicle control, t-test. **(C)** Preliminary cytotoxicity assay 2 results expressed as the mean number of colonies ±SE. *p ≤ 0.05 vs. vehicle control, t-test. **p ≤ 0.01 vs. vehicle control, t-test. **(D)** Cell Transformation assay (CTA) concurrent cytotoxicity (CC) results expressed as the mean number of colonies ±SE. *p ≤ 0.05 vs. vehicle control, t-test. **p ≤ 0.01 vs. vehicle control, t-test. Abbreviations: SE: standard error; DMSO: Dimethyl sulfoxide 0.5%; MCA: 3-Methylcholanthrene 4 μg/μL

In a preliminary cytotoxicity study, 13 concentrations ranging from 0.05 to 100 μg/mL, were explored through two clonal efficiency tests. Cells were treated for 24 h after seeding and exposed for 72 h (Figure 1). The tested chemicals exhibited toxic effects in the concentration range 10–100 μg/mL. The cells treated with higher concentrations exhibited extremely low colony-forming activity. These results were confirmed by CTA (Figure 1). The IC50 value was calculated through interpolation and estimated to be approximately 17 μg/mL.

### 3.2 Cell transformation assay

The effect of DEHP on the transformation frequency of BALB/c 3T3 A31-1-1 cells was assessed according to the protocol recommended by ECVAM (Sasaki et al., 2021a; Corvi et al., 2012; Tanaka et al., 2012; IARC, 2013; Mascolo et al., 2018).

**FIGURE 2:**
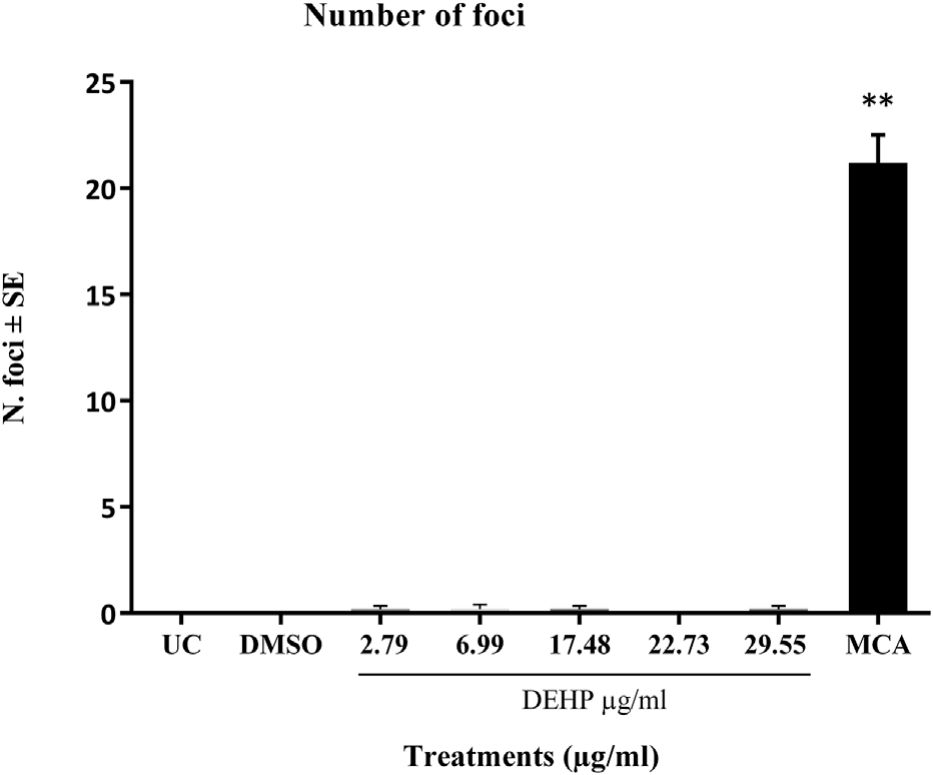
Cell Transformation assay (CTA) results expressed as the mean number of foci ±SE. **p ≤ 0.01 vs. vehicle control, t-test. Abbreviations: SE: standard error; DMSO: Dimethyl sulfoxide 0.5%; MCA: 3-Methylcholanthrene 4 μg/μL. Cells in the logarithmic growth phase were seeded at a density of 1 × 10^4 cells/60 mm dish, with 10 dishes per treatment, in M10F culture media. After 24 h, the cells were exposed to the test item for 72 h. Untreated cells and solvent-treated cells served as the negative controls, while cells treated with MCA represented the positive controls. From day 8 post-seeding, the culture medium was replaced twice a week with DF2I2F containing a low concentration of FBS (2%) and insulin (final concentration 2 μg/mL). At 24 days post-seeding, no further medium changes were observed. At day 31-32 post-seeding, the plates were fixed with methanol and stained with 0.04% Giemsa. The occurrence of transformed Type III foci was assessed using an optical microscope, characterized by deep basophilic staining, random cell orientation, dense multilayering of cells, and invasion into the surrounding contact-inhibited monolayer (Sasaki et al., 2012b).

The positive control MCA (4 μg/mL) induced a statistically significant increase in the number of transformed type III foci, which were almost absent in untreated and solvent-treated cells (Figure 2).

DEHP treatment did not significantly increase the formation of malignant foci in BALB/c 3T3 A31-1-1 cells (Figure 2).

Based on these criteria, DEHP can be classified as negative on CTA.

### 3.3 Molecular data analysis

Based on GeneSpring analysis using the unpaired t-test (*p* < 0.05) and Benjamini-Hochberg multiple test correction, 13.164 genes were identified after the analyses, of which 7.870 were upregulated and 5.294 were downregulated. Next, using the unpaired t-test (*p* < 0.05) and Bonferroni multiple test correction, 334 differentially expressed genes were identified, of which 240 were upregulated and 94 were downregulated. The latter gene list constitutes a subset of the former because all genes are common (data not shown; available at https://www.ebi.ac.uk/biostudies/arrayexpress).

The first differentially expressed transcriptome dataset (*n* = 13.164 genes) was imported into the MetaCore™ integrated software suite and functionally processed for functional enrichment by “Pathway Map” ontologies using the Functional Ontology Enrichment tool. A fold-change threshold of ±1.5 was applied.

Pathway enrichment analysis helps highlight mechanistic insights into gene lists generated from genome-wide transcriptomic experiments. The Pathway map Ontology Enrichment Analysis scored and sorted 5,573 network objects and more than 200 perturbed pathway maps with false discovery rate (FDR) < 0.05 (Supplementary Table S2).

The filter Pathway Maps using the category MetaCore option were used to split the maps into four categories: metabolic maps, regulatory maps, toxicity processes, and disease maps (Supplementary Table S2; Supplementary Figures S1–S4). Each pathway map could be related to more than one category.

Regulatory maps resulted in the most represented category, which was analyzed in the discussion with particular attention to the top most significant pathways (Supplementary Figures S1–S3; Supplementary Table S2).

**FIGURE 3:**
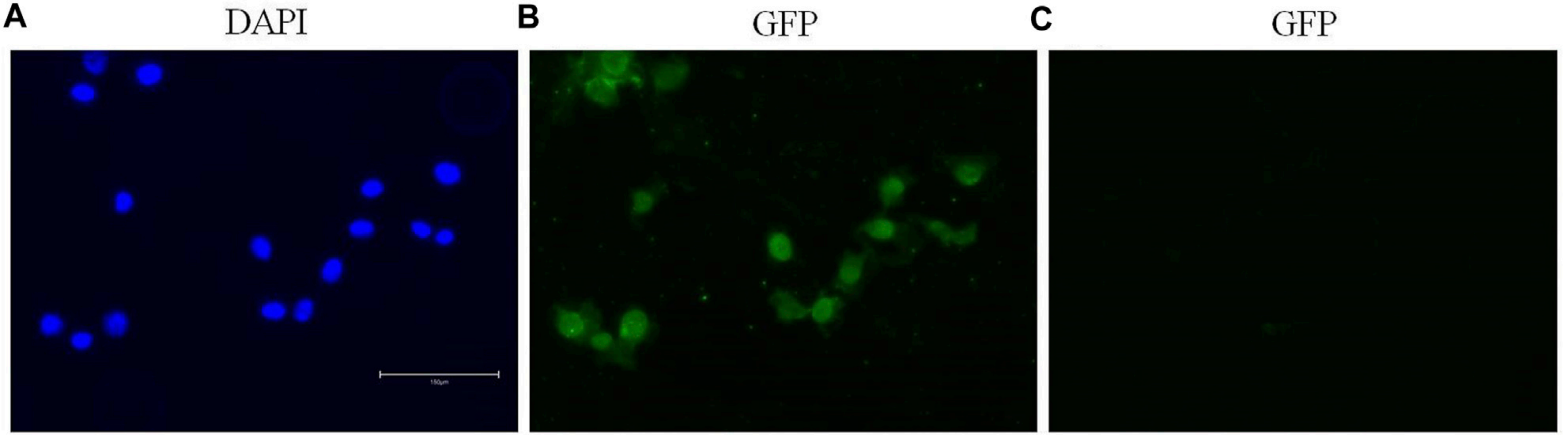
The first ten pathway maps with a False Discovery Rate (FDR) less than 0.05, as identified using the “Filter Pathway Maps by Category” function in MetaCore, categories: “Tox processes,” modulated by DEHP treatment in the BALB/c 3T3 A31-1-1 cell model. Produced with MetaCore.

A focus on the Tox process-modulated map was proposed to analyze the dataset in view of toxicogenomics and the modulation of drug-metabolizing nuclear receptors and enzymes (Figure 3; Table 2).

**TABLE 2:** Differentially expressed genes within the pathway “PXR-mediated direct regulation of xenobiotic metabolizing enzymes/Rodent version,” are involved in the regulation of lipid homeostasis.

Gene symbol	FC	Enzymatic activity
CYP11A1	1.61	Cytochrome P450 (Phase 1 metabolic enzyme)
CYP27A1	1.61	Cytochrome P450 (Phase 1 metabolic enzyme)
CYP2C19	1.58	Cytochrome P450 (Phase 1 metabolic enzyme)
CYP2C8	23.92	Cytochrome P450 (Phase 1 metabolic enzyme)
CYP2C9	4.21	Cytochrome P450 (Phase 1 metabolic enzyme)
CYP3A5	1.60	Cytochrome P450 (Phase 1 metabolic enzyme)
CYP3A7	1.53	Cytochrome P450 (Phase 1 metabolic enzyme)
ELOVL6	−1.56	Elongation of very long chain fatty acids protein 6 (Lipid metabolism)
MDR1	3.17	Multidrug resistance protein 3 Protein (Renal secretion)
SLC21A7	−2.48	Solute carrier organic anion transporter family member 1A5 Protein (Cholehepatic circulation of bile acids)
UGT1A1	2.58	UDP-glucuronosyltransferase (Phase 2 metabolic enzyme)
UGT1A6	5.40	UDP-glucuronosyltransferase (Phase 2 metabolic enzyme)

The gene modulation observed after 72 h of exposure is reported in Table 3, where the top 10 modulated gene pathways and the genes involved in the modulation of each pathway are shown.

**TABLE 3:** Pathway map ontology enrichment analysis: Top 10 statistically significant pathway maps modulated by DEHP treatment (19.7 μg/mL) for 72 h in BALB/c 3T3 A31-1-1 cells.

PathwayMap	Regulated objecks	*p*-value	FDR	Biological interpretation	Upregulated genes	Downregulate genesd
Apoptosis and survival_Granzyme A signaling	18/41	3.971E-10	3.953E-07	Apoptosis	IL-6, Collagen IV, Fibronectin, Histone H1, Histone H2B	APEX, HSP70, IFN-alpha, Lamin B1, HMGB2, NDPK A, Histone H3, hnRNP A2, PARP-1, TLR4, SET, hnRNP C, hnRNP A1
Oxidative stress_ROS signaling	30/108	5.306E-10	3.953E-07	Oxidative stress	PTEN, TfR1, p300, HES1, ADAM17, VEGF-ATXNIP (VDUP1), ATM, NOTCH1 (NICD), c-Abl, PLK3 (CNK), GADD45 alpha, p38 MAPK, MDM2, PKC, IL-6, NRF2, COX-2 (PTGS2), ERK1/2	ACACA, APEX, Bax, Cyclin B1, E2I, FASN, iNOS, GRP75, PUMA, PKA-reg (cAMP-dependent), SCD
Immune response_IL-6 signaling via JAK/STAT	21/71	6.281E-08	3.119E-05	Immune response inflammation	TEC, CDK4, Rac1, p300, ADAM17, VEGF-A, c-Fos, AP-1, IL-6 receptor, IL6RA, sIL6-RA, SHP-2, ADAM10, JunB, gp130, IL-6, CDK6, COX-2 (PTGS2)	p18, iNOS, RacGAP1
Signal transduction_RANKL-dependent osteoclast differentiation	21/81	7.329E-07	2.730E-04	Immune response	TEC, NF-AT1(NFATC2), PI3K reg class IA, TCIRG1 (Atp6i), OSCAR, Syndecan-4, IFRD1, c-Fos, AP-1, MITFFra-1, p38 MAPK, CDK6, Calcineurin A (catalytic)	ATF-4, Calmodulin, iNOS, CREB1, MMP-9, PPA5, Rac1
DNA damage_ATM/ATR regulation of G2/M checkpoint: cytoplasmic signaling	16/51	9.653E-07	2.877E-04	DNA damage	MLCP (cat), PP1-cat, Cul1/Rbx1 E3 ligase, PP2A regulatory, Brca1, beta-TrCP, ATM, c-Abl, ATR, GADD45 alpha, p38 MAPK, p38gamma (MAPK12), ERK2 (MAPK1)	Cyclin B1, Histone H3, UBE2C
Signal transduction_Calcium-mediated signaling	19/72	1.838E-06	4.564E-04	cytoskeleton remodelling	MLCP (cat), p300, Tiam1, c-Fos, ACTA2, p38 MAPK, PKC, CaMKK, CaMKK2, MUNC13, Calcineurin A (catalytic), COX-2 (PTGS2), ERK1/2	MMP-9, NURR1, Calmodulin, CREB1, PPA5, Rac1
Signal transduction_mTORC1 downstream signaling	17/60	2.191E-06	4.664E-04	Metabolism Autophagy	Rictor, ATG13, eIF4A, VEGF-A, PPARγ, eEF2K, PDIP46, MDM2	ACSL3, ATF-4, CBP80, eIF4B, MTHFD2, YY1, MVK, RPS6, SCD
Immune response_IL-6 signaling via MEK/ERK and PI3K/AKT cascades	19/74	2.875E-06	4.760E-04	Immune response inflammation	TEC, PI3K reg class IA (p85), PI3K reg class IA, ADAM17, Proepithelin, c-Fos, IL-6 receptor, IL6RA, sIL6-RA, SHP-2, PLC-beta1, ADAM10, JunB, gp130, IL-6, ERK1/2	Bax, RPS6, CREB1
G-protein signaling_Rac1 activation	19/74	2.875E-06	4.760E-04	cytoskeleton remodelling	PI3K reg class IA, Rho GTPase, DOCK4, Tiam1, KIDINS220, DOCK7, PI3K reg class IB (p101), Dcc, CaMK II alpha, ALS2, Semaphorin 3A, AF-6, CaMKK2, EPS8	G-protein beta/gamma, Rac1-related, Rac1, SHANK, TrkB
Eosinophil adhesion and transendothelial migration in asthma	18/68	3.259E-06	4.855E-04	Adhesion Inflammation	P-selectin, MGF, alpha-1/beta-1 integrin, C3aR, PLAU (UPA), Histamine H4 receptor, CD67, Collagen IV, Fibronectin, alpha-6/beta-1 integrin, p38 MAPK, PKC, ERK1/2	CCL5, MMP-9, C3a, Calmodulin, Eotaxin

The analysis was conducted using the Metacore™ software V6.34 (Clarivate Analytics; https://portal.genego.com).

### 3.4 Immunofluorescence staining

**FIGURE 4:**
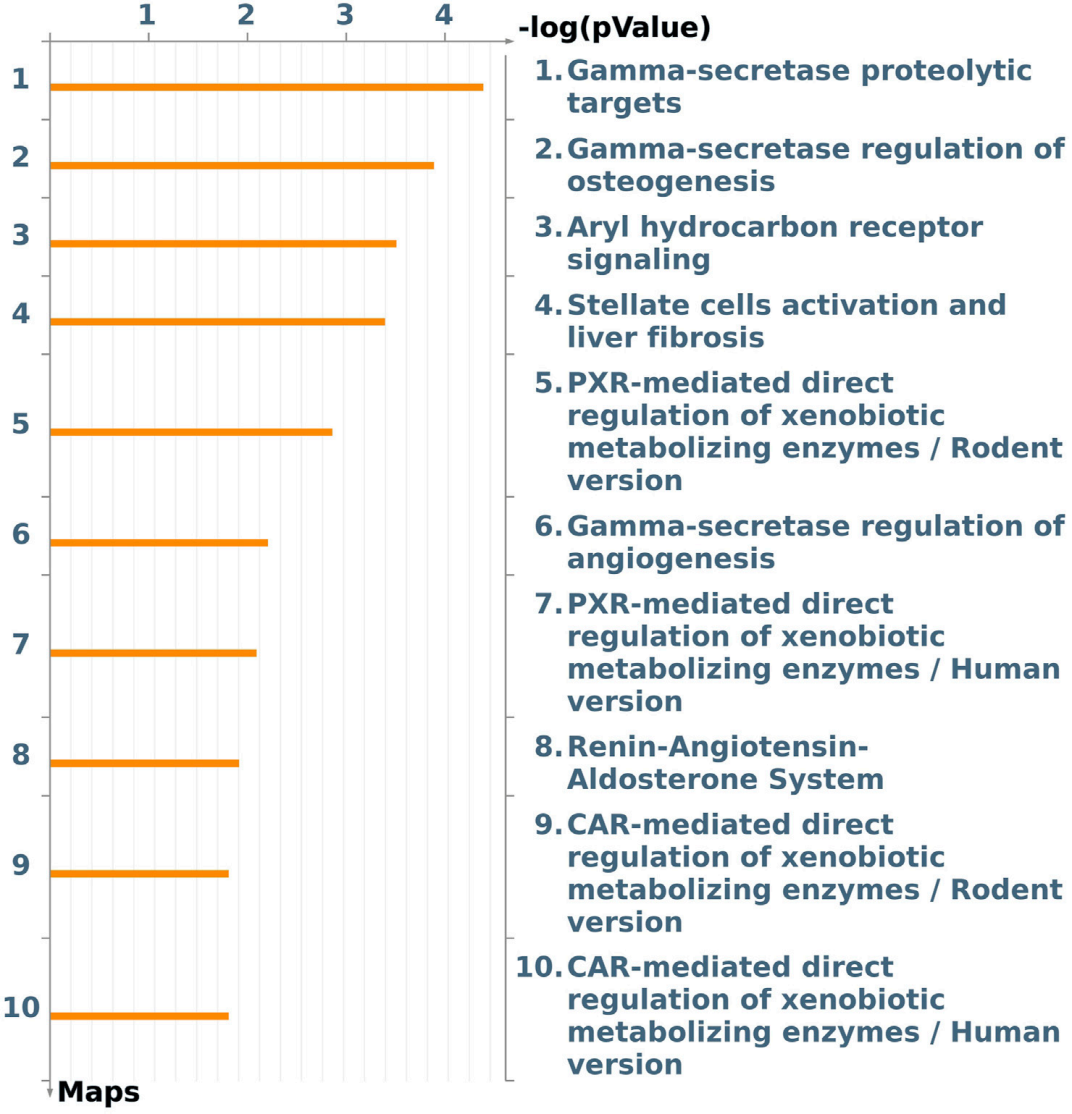
Expression of PPARα in BALB/c 3T3 clone A31-1-1 cells. Images were captured using the EVOS M5000 imaging system ×20 objective. **(A)** DAPI filter for the visualization of Hoechst staining. **(B)** GFP filter for the visualization of Alexa Fluor^®^ 488 (anti-PPAR alpha antibody, Abcam ab61182). **(C)** GFP filter: negative control, cells treated with the secondary antibody only.

Immunofluorescence staining permitted the detection and visualization of PPARα protein in the nuclear compartment of BALB/c-3T3 A31-1-1 cells (Figure 4).

## 4 Discussion

The primary objective of this study was to evaluate the potential of DEHP in standard CTA, using A31-1-1 BALB/c-3T3 cells.

Early attempts to develop omics-based CTA models revealed that most, if not all, key events and biological processes leading to oncotransformation are common to all the three current models of CTA. However, the gene transcript enrichment for each process highlights the ability of each model to emphasize different aspects of the process (Colacci et al., 2023). Primary SHE cells allow the identification of several gene signatures related to cytoskeleton remodeling, the first necessary condition for malignant changes, and events related to cell cycle control and senescence bypassing. Bhas 42 CTA is better suited for investigating mitogenic signals downstream of the activation of key oncogenes associated with RAS gene activation. BALB/c 3T3 CTA is an excellent model for investigating the role of inflammasomes and immune-mediated inflammation in malignancy through epithelial-mesenchymal transition, which is recognized as the committed step at the tissue level that marks dysplasia progression and acquisition of invasive properties. Moreover, transcriptomic analysis applied to CTA revealed the activation of receptor-mediated pathways involved in metabolic processes that are crucial for both the bioactivation and detoxification of chemicals. Specifically, the BALB/c 3T3 CTA has been reported to be a suitable model for elucidating the role of the aryl hydrocarbon receptor (AhR) in the activation and detoxification of xenobiotics. Therefore, we selected this model to investigate the molecular initiating events that drive DEHP-induced toxicity, and the early molecular events that are possibly related to DEHP toxicity.

DEHP has been extensively tested in CTAs using various protocols and cell models over time (Supplementary Table S1), revealing predominantly positive outcomes in SHE CTA and negative results in BALB/c 3T3 CTA (Supplementary Table S1). Notably, the conventional SHE CTA protocol employed 0.2% (v/v) DMSO as the vehicle, and the exposure duration for the cells in this assay was 7 days. It is important to acknowledge that many BALB/c 3T3 assay studies have been conducted more than 20 years ago, exhibiting significant variability in the experimental conditions and vehicles used. Furthermore, numerous studies have been conducted at DEHP concentrations that surpass their solubility limits, complicating their interpretation. Additionally, despite the use of organic solvents such as DMSO, F68 Pluronic, and acetone as vehicles, several studies have emphasized the poor miscibility and solubility of the test item. Finally, the original CTA protocols have undergone substantial modifications and amendments over the years, potentially influencing observed outcomes (Colacci et al., 2023).

Therefore, one of the objectives of this study was to explore key factors concerning experimental conditions that may influence the ultimate outcome when working with poorly soluble chemicals in order to refine the CTA experimental protocols.

In this study, a particular focus was placed on the dissolution of DEHP in the cell media. The test chemical stock solution was prepared by dissolving DEHP in DMSO, and the stock solution was diluted in the culture medium at various concentrations. The final concentration of DMSO in the cell medium was 0.5% (v/v), which was preferred over the more typical 0.1% (v/v) concentration to ensure homogeneous distribution of DEHP, as previously recommended (Sasaki et al., 2012a).

The results of clonal efficiency tests revealed a greater cytotoxic effect of DEHP in BALB/c 3T3 A31-1-1 cells than in previous *in vitro* studies (Supplementary Table S1). A concentration-dependent reduction in colony formation was observed at relatively low concentrations, beginning at 10 μg/mL, corresponds to 10.20 μL/mL.

We hypothesized that the higher concentration of DMSO used in this experiment would enhance the bioavailability of DEHP in cells, resulting in a more significant effect.

Notably, no increase in the cell transformation rate associated with DEHP exposure was observed in this study, which is consistent with the findings of previous studies that used the same CTA model.

It is widely recognized that results obtained from SHE and BALB/c 3T3 cell transformation assays can vary significantly, and various key events and biomarkers have been identified for each model (Colacci et al., 2023; Benigni et al., 2012). Moreover, it has been suggested that SHE may be more sensitive to a broader range of carcinogenic types than other cell transformation assays, as it detects more basic and nonspecific mechanisms and earlier stages of cell transformation (Colacci et al., 2023).

However, the precise mechanism by which DEHP induces malignant transformation in SHE cells remains unclear, despite evidence suggesting that it proceeds independent of PPAR activation (Tsutsui et al., 1993; Landkocz et al., 2011; Colacci et al., 2023).

More specifically, DEHP, MEHP, clofibrate, or WY-14,643 did not induce peroxisome proliferation in the SHE model when treated in the absence of exogenous metabolic activation, but DEHP was still able to induce cell transformation (Isenberg et al., 2000; Isenberg et al., 2001).

Furthermore, it should be noted that the inhibition of gap junctions intercellular communication (GJIC), peroxisomal β-oxidation and enhanced cell replication in rodent livers following DEHP, feeding have been identified as reversible effects. These effects persisted throughout the treatment period but were reversed upon discontinuation of the treatment. (Isenberg et al., 2000; Isenberg et al., 2001). Additionally, the inhibition of GJIC has been described as a transient effect in the SHE cell model (Cruciani et al., 1997).

It is reasonable to hypothesize that the unfavorable results observed in the BALB/c-3T3 CTA could be attributed to the shorter duration of chemical exposure compared to the standard 7 days exposure required in the SHE CTA. Indeed, DEHP failed to induce SHE cell transformation after a 24 h period (LeBoeuf et al., 1996). This difference may be noteworthy, because the mechanisms involved may be transient. It is important to mention that we carried out transcriptomic experiments on cells that had been treated with a toxic concentration of DEHP (19.7 μg/mL for 24 h and 72 h). This concentration is close to the half-maximal inhibitory concentration (IC50) value.

### 4.1 Regulation pathway maps

The pathway map with the lowest False Discovery Rate (FDR) is the “Protein folding and maturation\_Amyloid precursor protein processing” pathway (pathway #1; FDR 7.303e-8, 25 modulated network objects out of 50). This pathway involves the amyloid precursor protein (APP) processing scheme, with APP mRNA being the primary gene involved, exhibiting a Fold Change (FC) of 1.81. Other genes involved in this pathway include matrix metalloproteinase 9 (MMP9; FC −3.14) and beta-secretase 2 (BACE2; FC 1.58) (Supplementary Table S2).

#### 4.1.1 APP pathway

APP is a type 1 transmembrane glycoprotein that plays a critical role in neural transmission, neuronal homeostasis, and development. Alternative splicing generates APP mRNAs that encode several isoforms with tissue-specific and physiological functions. APP has been extensively studied as a precursor of amyloid β neurotoxic peptides in Alzheimer’s disease. APP is particularly expressed in neuronal tissues and its expression is upregulated following brain injury (Liang et al., 2020).

Exposure to DEHP during early life or pregnancy has been linked to increased amyloid-β toxicity in *Caenorhabditis elegans* (Yen et al., 2021). Furthermore, animal and epidemiological studies have demonstrated a positive correlation between DEHP exposure in early childhood or maternal exposure during pregnancy and various neuropathologies and neurobehavioral diseases, suggesting a neurotoxic action of DEHP. This neurotoxicity has primarily been attributed to cellular oxidative damage, apoptosis, and ion channel imbalance (Liu et al., 2023).

APP has been found to be expressed in non-neuronal tissues and overexpressed in several types of cancer (Lee et al., 2021). Additionally, multiple fragments generated by the proteolytic processing of APP have been implicated in the regulation of cholesterol metabolism and may directly influence Low Density Lipoprotein Receptor (LDLR) expression (Wang et al., 2014).

Despite conducting an enrichment analysis, we did not identify any significantly modulated pathways specifically related to lipid metabolism and trafficking. However, we observed modulation of several genes related to these cellular processes, which will be discussed later. For example, we noted modulation of the LDLR (FC −1.79) and LDLR-related protein 1 (LRP1, FC −2.01).

BACE2, along with BACE1, has been extensively studied in the context of Alzheimer’s disease, as both enzymes are responsible for processing APP into neurotoxic Aβ peptides. Conversely, BACE2 is ubiquitously expressed and can cleave APP at a site different from that of BACE1, producing non-neurotoxic peptides. BACE2 has also been linked to type 2 diabetes and tumor progression (Farris et al., 2021).

Interestingly, abnormal APP metabolism in the pancreas has been linked to type 2 diabetes, and recent epidemiological evidence suggests a strong association between diabetes and Alzheimer’s disease (Hamzé et al., 2022).

#### 4.1.2 POMC pathway

The second pathway map pertains to a distinct peptide processing mechanism, specifically, “Protein folding and maturation\_POMC (pro-opiomelanocortin protein) (pathway #2) (FDR 2.518e-5)”. POMC is a prohormone found in various tissues and undergoes extensive post-translational modifications, resulting in the generation of diverse sets of tissue-specific peptides that perform various biological functions (Raffin-Sanson et al., 2003). The most well-studied POMC polypeptide is the 29-Kd polypeptide, which is post-translationally processed in the pituitary gland to form biologically active peptides, such as adrenocorticotropin (ACTH), endorphins (α-, β-, γ-EP), and melanotropins (α-, β-, γ-MSH). These peptides are involved in the regulation of the melanocortin pathway in response to leptin and insulin. The central melanocortin system plays a key role in regulating energy metabolism and body weight homeostasis, as evidenced in numerous recent studies (Li et al., 2023).

The “POMC, alpha-MSH, and AGRP in the regulation of food intake and energy expenditure in obesity in the hypothalamus” pathway map (pathway #149; FDR 1.118e-2; 13 modulated network objects out of 43) is highlighted for significant modulation. This pathway includes the overexpression of melanocortin receptor 4 (MCR-4, FC 1.69) and agouti-related neuropeptide (Agrp, FC 1.67), which act as antagonists of melanocortin receptor signaling, as well as the downregulation of brain-derived neurotrophic factor (BDNF, FC −1.62) (Supplementary Table S2).

These findings support the effects of neurotoxic and endocrine disruptors such as DEHP at the hypothalamic level in rodents (Lv et al., 2016). Wang et al. (2014) Lv et al. focused on the mechanisms underlying the impact of DEHP on the pathogenesis of obesity and hypothyroidism as well as the relationship between the two conditions, supported by the downregulation of thyroid hormone receptor beta (TR-beta) and Retinoid X receptors (RXR) genes in DEHP-treated C3H/He mice.

Our data revealed the modulation of two receptors for thyroid hormones: TR-beta and thyroid hormone receptor alpha (TR-alpha) [FC 2.95 for Thrb(A\_51\_P388835) and 1.58 for Thrb(A\_52\_P532559)] for TR-beta, and FC 1.66 for TR-alpha). It is important to note that, in the first two pathway maps, several network objects were derived from only one or a few modulated transcripts. Both pathways are characterized by upregulation of peptides, which have been studied for their potential “bridging roles” in metabolic regulation and neurophysiological implications.

#### 4.1.3 FGFR pathway

The third pathway map in the list is “The Signal transduction\_Nuclear FGFR1 signaling” pathway #3; FDR 2.418e-5. Fibroblast growth factor (FGF) family signaling through the receptor tyrosine kinase FGF receptors (FGFR) regulates many cellular processes and plays essential roles in the early stages of embryonic development. In contrast to the first two pathway maps, this map consisted of 88 genes, 30 of which were modulated by DEHP treatment at 19.7 μg/mL for 24 h. FGF1 has emerged as a key regulator of bile acid, lipid, and carbohydrate metabolism, and in this pathway, FGFR1 is upregulated (FC 1.5). (Supplementary Table S2).

It is important to mention that FGFR1 has been proposed as a potential regulator of adipogenesis and may contribute to obesity by modulating the number of fat cells.

Although there was a slight upregulation of FGFR1, the overall trend of this molecular signaling appeared to be inhibited, as several downstream target genes were downregulated, whereas diverse downstream FGF-inhibited targets were upregulated.

Previous studies have shown that two sets of growth factors are necessary for efficient stimulation of DNA synthesis in murine BALB/c 3T3 fibroblasts. The first set includes platelet-derived growth factor (PDGF) and FGF, rendering the cells “competent” to enter the S phase. Competent cells respond to a second set of growth factors, including epidermal growth factor (EGF) and insulin-like growth factor-1 (IGF-1), which allows the “progression” of cells into the cell cycle (Jones and Kazlauskas, 2001).

In our dataset, some FGF-related transcripts resulted in upregulation, as well as the EGFR ligand EGF, which showed an increase of 1.59 for Egf (A\_55\_P2733187) and 1.64 for Egf (A\_55\_P2822952). On the other hand, some PDGF-related transcripts resulted in downregulation, including PDGF-B, which showed a decrease of FC −2.43 for Pdgfb (A\_55\_P2047310) and FC −2.32 for Pdgfb (A\_55\_P2733467), and PDGF receptor subunit (PDGF-R-alpha), which decreased by FC −2.00 for Pdgfra (A\_51\_P345649), −1.71 for Pdgfra (A\_55\_P2734892), and −2.03 for Pdgfra (A\_55\_P2735715). Similarly, the insulin-related transcripts, Insulin substrate receptor-1 (ISR-1), and the Insulin-like growth factor 1 receptor Protein (IGF-1 Receptor), decreased by FC −2.21 for ISR-1, and FC −1.69 for Igf1r (A\_52\_P668647) and −1.72 for Igf1r (A\_55\_P2804885).

Notably, FGF1 plays a role in adaptive adipose remodeling (Wang et al., 2020; Sancar et al., 2022; Hamzé et al., 2022). FGF1 expression in adipose tissue is regulated by PPARγ and mice lacking FGF1 develop a more aggressive diabetic phenotype in response to dietary challenges (Sancar et al., 2022).

Additionally, among the extensively modulated “regulatory pathway maps,” the regulation of metabolic pathways was highlighted using a MetaCore filter. Interestingly, among the last category of pathway maps, three significantly modulated pathways were highlighted: 1) “signal transduction_ WNT/β-catenin signaling in tissue homeostasis” (pathway #19) (FDR 2.010e-4; 17 modulated network objects out of 42); 2) “regulation of metabolism of GLP-1 signaling in beta cells” (pathway #34); (FDR6.727 e-4; 26 modulated network objects out of 91); and 3) “regulation of metabolism: glucocorticoid receptor signaling in glucose and lipid metabolism” (FDR 5.027e-2; 17 modulated network objects out of 80).

Overall, these transcriptome results support the toxic action of DEHP on cell metabolism, leading to impaired insulin signal transduction and the deregulation of glucose utilization and lipid synthesis.

DEHP causes obesity and hypothyroidism in both humans and rodents and induces lipid metabolism disorders, liver toxicity, and adrenocortical dysfunction (Tickner et al., 2001; Lv et al., 2016; Zhang et al., 2023). Evidence has shown that exposure to DEHP increases blood glucose levels, impairs energy metabolic balance, induces insulin resistance, and leads to prediabetes (Dales et al., 2018).

#### 4.1.4 Possible involvement of PPAR regulation

Although the MetaCore pathway map “Regulation of lipid metabolism_PPAR regulation of lipid metabolism” did not exhibit significant modulation in this enrichment analysis (FDR 4.901e-1), several genes associated with PPARα signaling, fatty acid metabolism, and beta-oxidation were modulated in this experiment.

Fatty acid-binding protein 1 (Fabpl; FC 1.61) is upregulated, potentially facilitating fatty acid delivery to the nucleus and enhancing ligand-mediated transactivation of PPARα by directly binding to PPAR agonists (Hughes et al., 2015).

Additionally, the long-chain fatty acid transporter (Slc27a1, also known as FAT1; FC 2.32) was upregulated, suggesting the involvement of fatty acid transmembrane transporter activity, long-chain fatty acid import into cells, and the positive regulation of triglyceride biosynthetic processes.

L-bifunctional enzyme (Ehhadh; FC 1.59), also known as peroxisomal bifunctional enzyme protein, is part of the classical peroxisomal fatty acid β-oxidation pathway and is induced by PPARα activation. Long-chain-fatty-acid-CoA ligase 1 [ACLS1; FC 1.58 for Acsl1(A_51_P496432) and 1.51 for Acsl1(A_52_P597618)] was observed to convert long-chain fatty acids to acyl-CoA products via an ATP-dependent pathway and could be induced by both PPARα and PPARγ. Furthermore, the 3-ketoacyl-CoA thiolase peroxisomal protein [FC 1.94, Acaa1a (A_52_P155990) and 1.74 for Acaa1a (A_55_P2076580)], located upstream of or within fatty acid beta-oxidation and found in the mitochondria, exhibited modulation. The peroxisomal acyl-coenzyme A oxidase 3 protein (FC 1.81) has been implicated in the desaturation of 2-methyl-branched fatty acids in peroxisomes. Additionally, the carnitine palmitoyltransferase 1A (CPT-1A; FC 2.00), a key enzyme in the positive regulation of fatty acid beta-oxidation and insulin secretion regulation, and acyl-CoA synthetase long-chain family member 1 (ACSL1; FC 1.58 for A_51_P496432 and 1.51 for A_52_P597618) has been identified. CPT-1 was identified as a PPARα activation marker in DEHP-exposed mice (Lv et al., 2016).

PPARα-related toxicity induced by DEHP has been described as a series of events starting with receptor activation, resulting in peroxisome proliferation, induction of peroxisomal proteins, elevated fatty acid metabolism, increased cell proliferation and decreased apoptosis, production of reactive oxygen species, oxidative DNA damage, and inhibition of gap junctional intercellular communication. These events are associated with DEHP-induced hepatocarcinogenesis in rodents (Ito et al., 2007; Corton et al., 2014; Rajesh and Balasubramanian, 2014). It has been suggested that DEHP can stimulate the activation of PPARγ, leading to oxidative stress, downregulation of insulin receptor and GLUT4 protein expression, and disruption of insulin signaling (Mariana and Cairrao, 2023).

Initially, we confirmed PPARα expression in our cellular model to rule out the possibility that the negative outcome was due to the absence of what is commonly regarded as the primary receptor of DEHP.

In this study, we discovered the activation of certain peroxisomal proteins and regulation of genes involved in fatty acid metabolism. Furthermore, there was an indication of reduced insulin signaling, with an intriguing increase in the mRNA of the insulin-regulated glucose transporter GLUT4 (Slc2a4, Solute carrier family 2, facilitated glucose transporter member 4 Protein, FC 2.04) compared with the control.

However, the precise mechanisms governing cell survival and proliferation remain unclear. In the subsequent pathway map analysis, we highlighted the regulation of proliferation and extracellular matrix reorganization signaling. The pathway map “TGF-beta signaling via SMADs in breast cancer” (pathway #4) (FDR 4.910e-5) pertains to TGF-beta signaling and its role in breast cancer and its associated metastases. We observed a general reduction in the expression levels of these factors.

This finding suggests the potential inhibition of TGF-β signaling. Additionally, we identified other pathway maps connected to TGF-β signaling, including maps #8, #12, #17, and #31. Several transcripts associated with proteins involved in extracellular matrix reorganization were found to be downregulated, such as MMP-2 (FC −1.55), MMP-9 (FC −3.14), Stromelysin-1 (FC −9.44), and MMP-13 (FC −5.34). Notably, some transcription factors that regulate the transcription of these proteases were found to be downregulated in our study. Moreover, the gene network can be analyzed using the pathway “Signal transduction_PDGF signaling via MAPK cascades” (pathway #5) (FDR 5.293e-5), which appears to be inhibited because the upstream factors PDGF-B (FC −2.43) and PDGFR-alpha (FC −2.03) were both downregulated. Additionally, Hyaluronan synthase 2 (HAS2) and hyaluronan synthase 1 (HAS1) were modulated, HAS2 was downregulated (FC −3.44), and HAS1 was slightly upregulated (FC 1.58).

The relationship between LAMA3 (Epiligrin, FC −2.27) and the extracellular matrix remodeling process was confirmed. Thrombospondin 1 [FC −3.67 for Thbs1 (A\_55\_P2746459) and −3.88 for Thbs1 (A\_65\_P13588)] is also in agreement with this process, as observed in the modulated pathway map “CHDI\_Correlations from Discovery data\_Causal network (positive)” (pathway #6) (FDR 5.293e-5). In this map, ephrin signaling was also modulated. Ephrins and their receptors play important roles in regulating cell migration and adhesion, with Ephrin-B receptors and Ephrin-B being downregulated (respectively FC −1.80 for the receptor and FC −1.76 for Efnb1, -2.78 for Efnb2, and −1.74 for Efnb3 ligands) (pathways #6, #14, #20, #28, #54, and #104). The modulation of “cytoskeleton remodeling and regulation of actin cytoskeleton organization by the kinase effectors of Rho GTPases” (pathway #18) (FDR 1.337e-4) is related to this issue.

The downregulation of these metalloproteinases can also be observed within the pathway map “Immune response,_IL-17 signaling” (pathway #10) (FDR 9.344e-5), which shows an upregulation of the cytokines IL-21, IL-17, and IL-17R, which are involved in the differentiation, maintenance, and expansion of Th17 cells, and play an important role in regulating oxidative stress and inflammation (Supplementary Table S2).

Based on these findings, we conclude that DEHP treatment affects cell-cell adhesion and cell-matrix adhesion. Treatment appears to increase cell-cell contact and cell-matrix adhesion. Moreover, the extracellular matrix is reinforced through the overexpression of Col1A2, which increases collagen and E-cadherin, which act as cell–cell adhesion molecules by connecting with cytoplasmic β-catenin to form cadherin/catenin complexes. Recently, it was shown that IGF-1 is inversely associated with E-cadherin expression in various types of cancers (Zeljkovic et al., 2020).

In addition, gene expression analysis in SHE cells exposed to DEHP revealed an unexpected outcome regarding the cell-matrix adhesion processes. Specifically, a temporary increase in cell adhesion was observed after 5 h of exposure to all the tested doses (Landkocz et al., 2011). It has also been proposed that TGF-β signaling is regulated (Landkocz et al., 2011).

Utilizing the Pathway filter option and focusing on regulatory pathways pertaining to apoptosis and survival, it was noted that modulation of “signal transduction\_WNT/β-catenin signaling in tissue homeostasis” (pathway #42) (FDR = 2.010e-4) occurred. Several other significantly modulated pathway maps further support the modulation of WNT signaling. Activation of the canonical Wnt/β-catenin signaling pathway is influenced by both ligand and receptor contexts. In the current experiment, several WNT ligands and receptors were scored as deregulated: 1.60 for Wnt3a (A\_51\_P210970), −2.46 for Wnt5b (A\_55\_P1984976), −1.54 for Wnt7b (A\_52\_P231691), and 2.39 for Wnt9a (A\_55\_P2032147). WNT ligands bind to Frizzled receptors [FC 2.25 for Fzd4 (A\_51\_P361220) and 2.38 for Fzd4 (A\_66\_P132734)], which activate signaling via β-catenin and SNAIL1 (FC −1.75). After translocation to the nucleus, β-catenin regulates target gene expression via activation of several gene targets, including Lef-1(FC 2.54), TCF7 (FC 1.58), and TCF7L2 (FC −1.60).

It is essential to emphasize that the transcriptional profile described so far reflects gene modulation at 24 h. It may be necessary to expose cells for an extended period to identify genes associated with more significant cellular disruptions, including perturbation of metabolic pathways and cellular stress (Poitou et al., 2022).

### 4.2 Inflammation and immune responses

The significantly altered pathway maps included several pathways related to cytokine production, inflammation, and immune response. These pathways include “Immune response\_Histamine H1 receptor signaling in immune response” (pathway #9) (FDR 9.344e-5), “Immune response\_IL-17 signaling” (pathway #10) (FDR 9.344e-5), and “Immune response\_IL-6 signaling via JAK/STAT” (pathway #15) (FDR 1.168e-4). Additionally, pathways such as “Th2 cytokine- and TNF-alpha-induced profibrotic response in asthmatic airway fibroblasts/myofibroblasts” (pathway #13) (FDR 1.000e-4) and “TNF-alpha and IL-1 beta-mediated regulation of contraction and secretion of inflammatory factors in normal and asthmatic airway smooth muscle” (pathway #21) (FDR 2.361e-4) were also altered.

Within these pathways, several cytokine signaling factors, including IL-21, IL-17, IL-17R, IL-6R, INF-alpha, and IL-8RA, were upregulated. Notably, the transcription factor NFAT is overexpressed, which can induce the expression of several pro-inflammatory genes. The upregulation of transcripts may be linked to the activation of IP3 receptor signaling in the mitochondria where the IP3 receptor is upregulated. Activation of the IP3 receptor triggers the release of calcium from the endoplasmic reticulum into the cytosol, thereby activating calmodulin. Calmodulin activates Calcineurin A leading to NFAT activation. Additionally, upregulation of transcription factor Nuclear factor (Erythroid-derived 2)-like 2 (Nrf2), was observed. This gene encodes a transcription factor that regulates genes containing antioxidant response elements (ARE) in their promoters, many of which encode proteins involved in the response to injury and inflammation, including the production of free radicals (Saha et al., 2020). The overexpression of heme oxygenase supports the overexpression of Nrf2 as an anti-oxidative response.

Inflammation-related pathways are modulated by several downregulations, such as Nf-Kb (FC −1.65), COX-2 (FC −2.68), CCL20 (FC −3.43), VCAM [FC −2.41 for Vcam1(A\_51\_P210956) and −1.91 for Vcam1 (A\_52\_P520495)], IGF-1 receptor, and MMP-2 (FC −1.55) and MMP-9. Despite the upregulation of IL-6R, several downstream factors of this signaling pathway were downregulated. IL-6 activation can induce STAT3, leading to the initiation of the expression of activator protein 1 (AP-1, a complex of several subunits: FC −1.51 for Fos, FC −2.27 for Fosl2, FC −2.31 for Fosl2, FC −1.79 for Jun, FC −1.56 for Junb, and FC −1.54 for Junb) RUNX2, IL-RAP, c-Jun, and c-Fos factors, all related to inflammation and immune responses. On the other hand, IL-6 can also promote the activation of Mucin 4 and the angiogenic factor VEGF-A, which are both upregulated (FC 2.43 and 1.61, respectively).

These results support the activation of antioxidant and inflammatory signaling pathways in response to DEHP. The downregulation of related genes suggested downregulation of the NF-κB/AP-1 signaling pathway, which was supported by the upregulation of inhibitor of nuclear factor kappa B kinase regulatory subunit gamma (IKK-gamma, FC 1.51). This inhibition of signaling could be related to previously documented negative interference with PPARα activation. Indeed, PPARα activation can inhibit the nuclear translocation of the NF-κB/p65 subunit and reduce the phosphorylation of nuclear c-Jun/AP-1, thereby inhibiting the production of pro-inflammatory cytokines such as TNFα, IL-1β, Cox-2, and iNOS (Delerive et al., 1999; Xu et al., 2001; Korbecki et al., 2019).

### 4.3 Effects of DEHP on toxic pathways

An image was generated utilizing the “filter by Map Categories: Tox processes” function to display the top ten pathways in order of significance for toxic processes that may be induced by DEHP treatment in the BALB/c 3T3 A31-1-1 cell model (Figure 3).

This list of pathways focuses on the significant modulation of toxic processes.

The first three pathways in the list are all related to the gamma-secretase complex, which is involved in critical cellular processes through the cleavage of type I transmembrane proteins, such as Notch family proteins (FC 1.59) and APP (FC 1.88). The regulation and function of these proteins have been previously described. Additionally, presenilin mRNA, which is upregulated (FC 1.99), encodes a catalytic component of the gamma-secretase complex, and its essential functions in calcium homeostasis have been well-documented.

The Notch signaling pathway is involved in various processes including immune cell development, epithelial-to-mesenchymal transition, angiogenesis, mammary gland development, osteogenesis, and gastrointestinal cell differentiation. It also plays a crucial role in the regulation of the development of different tissues. In the context of this study, it was expected that the PPARα pathway would be modulated given that DEHP is a PPARα agonist. However, enrichment analysis revealed that Aryl hydrocarbon receptor (AhR) and Pregnane X Receptor (PXR) signaling pathways were also affected by DEHP treatment. Specifically, the “Aryl hydrocarbon receptor signaling pathway” (pathway #103) (FDR 4.720e-3) was the third most perturbed toxicity pathway in this study when considering the tox process pathway map list (Figure 3). This pathway includes 19 modulated genes out of a total of 53 modulated genes. The BALB/c 3T3 A31-1-1 cell model was found to have an active AhR signaling pathway, and immunofluorescence staining performed in the study showed that the cells were capable of expressing PPARα without any treatment, primarily in the nuclear compartment.

Some studies have suggested that DEHP may act as a weak agonist of AhR in human and rodent cell types, activating AhR signaling (Villard et al., 2007; Ernst et al., 2014; Zou et al., 2020; Ge et al., 2022; Hsieh et al., 2022).

Crosstalk between PPAR and AhR suggests that PPAR signaling regulates and activates AhR expression, ultimately downregulating estrogen synthesis by upregulating CYP1B1 and downregulating CYP19 signaling (Villard et al., 2007; Ernst et al., 2014). Both PPARα and PPARγ bind to estrogen response elements and act as competitive inhibitors, thereby affecting estradiol synthesis (Mu et al., 2000; Yanase et al., 2001; Fan et al., 2005; Benigni et al., 2012).

Phthalates also exhibit estrogen-like functions by binding to estrogen receptors and increasing estrogen synthesis by inducing aromatase expression (Chen et al., 2016; Zheng et al., 2023). In theory, AhR suppresses estrogen receptor 1 (ESR1, nuclear) signaling, recruiting both ESR1 and proteasomes, leading to ubiquitination and degradation of both AhR and ESR1 (Wormke et al., 2003). Additionally, AhR promotes the transcription of nuclear receptor-interacting protein 1 (RIP140), which inhibits ESR1 signaling (Augereau et al., 2006). Hsieh et al. (2022) also reported that DEHP mediates ER degradation via the AhR.

Notably, the 24 h exposure cells profile demonstrated an upregulation of ESR1 under treatment with 19.7 μg/mL DEHP (pathways #70, #103, #113, #167 FDR <0.05), along with gonadotropin-releasing hormone receptor (GnRH) (pathway #65) (FDR 1.879e-3). The altered expression of gonadotropin has also been linked to the disruption of AhR signaling by TCDD (Horling et al., 2011).

It is also intriguing to observe the modulation of pathway maps related to PXR signaling (Rodent/human version) (pathway #172) (FDR 1.291e-2). PXR is a nuclear receptor subfamily 1 group I member 2, pregnane X Receptor that is activated by a wide range of drugs, xenobiotics, and endogenous metabolites including steroids and bile acids. In specific cell types such as the liver and intestine, it serves as a “xenosensor” by regulating the expression of a network of genes involved in xenobiotic clearance. PXR is sequestered in the cytoplasm and translocates to the nucleus, where it forms a PXR/RXR-alpha complex with Retinoid X receptor alpha (RXRA) and binds to target gene promoters. Many plastic-associated endocrine-disrupting chemicals, such as BPA, BPB, and phthalates, have been reported to be potent agonists of the PXR (DeKeyser et al., 2011; Sui et al., 2012; Zhou, 2016; Helsley and Zhou, 2017; Sui et al., 2018).

Several studies have identified PXR as playing a role in maintaining lipid homeostasis and atherogenesis (de Haan et al., 2009; Cheng et al., 2012; Sui et al., 2012; Zhou, 2016; Helsley and Zhou, 2017; Sui et al., 2018; Gwag et al., 2019; Meng et al., 2019). For example, activating PXR through ligand-mediated means has been shown to raise plasma total cholesterol and atherogenic LDL levels in mice (Gwag et al., 2019; Meng et al., 2019).

In this context, the upregulation of the transcription factor hepatocyte nuclear factor 4 alpha (HNF4-alpha, FC 1.61) is highlighted as a key component of this pathway. HNF4-alpha is a crucial master transcription factor for the hepatic fat and bile acid metabolic pathways.

Notably, PXR and CAR regulate overlapping sets of genes encoding phase I- and II-metabolizing enzymes and transporters that are involved in xenobiotic detoxification and elimination. The DEGs associated with this pathway are listed in Table 2. Notably, the CAR pathway maps were not significantly modulated with an FDR of 6.229e-2 (Figure 3).

Additionally, the xenobiotic metabolizing systems induced by AhR, PXR, and CAR are involved in the metabolism of endogenous molecules such as steroids and thyroid hormones, including CYP3A. Induction of these systems may contribute to the endocrine disruptive activity of DEHP.

### 4.4 Sustained molecular signals after extended DEHP exposure and final remarks

We conducted a comprehensive analysis of the molecular signals after 24 h of DEHP exposure to identify the initial molecular events. Additionally, the transformation assay provided insights at the end of the 72 h exposure period (Table 3). Analysis of the results at this juncture revealed the amplification of signals observed at 24 h, confirming the involvement of the AhR receptor and the innate immune-mediated response initiated by IL-17 signaling and supported by IL-6, a pivotal interleukin in the inflammation pathway. Furthermore, signals indicative of PPARγ activation were observed (Table 3). Conversely, signals related to PPARα were diminished. A comparison of the top 10 modulated pathways at 24 h and 72 h is presented in Table 4.

**TABLE 4:** Comparison of molecular pathway modulation at 24 hours and 72 hours of DEHP exposure^a^.

#	Modulated pathway map at 24 h	FDR	Modulated pathway map at 72 h	FDR
1	Protein folding and maturation_Amyloid precursor protein processing (schema)	7.137E-08	Apoptosis and survival_Granzyme A signaling	3.953E-07
2	Protein folding and maturation_POMC processing	2.361E-05	Oxidative stress_ROS signaling	3.953E-07
3	Signal transduction_Nuclear FGFR1 signaling	2.361E-05	Immune response_IL-6 signaling via JAK/STAT	3.119E-05
4	TGF-beta signaling via SMADs in breast cancer	4.825E-05	Signal transduction_RANKL-dependent osteoclast differentiation	2.730E-04
5	Signal transduction_PDGF signaling via MAPK cascades	5.180E-05	DNA damage_ATM/ATR regulation of G2/M checkpoint: cytoplasmic signaling	2.877E-04
6	CHDI_Correlations from Replication data_Causal network (positive correlations)	5.180E-05	Signal transduction_Calcium-mediated signaling	4.564E-04
7	Signal transduction_CXCR4 signaling via MAPKs cascades	5.823E-05	Signal transduction_mTORC1 downstream signaling	4.664E-04
8	Development_Regulation of epithelial-to-mesenchymal transition (EMT)	9.200E-05	Immune response_IL-6 signaling via MEK/ERK and PI3K/AKT cascades	4.760E-04
9	Immune response_Histamine H1 receptor signaling in immune response	9.200E-05	G-protein signaling_Rac1 activation	4.760E-04
10	Immune response_IL-17 signaling	9.200E-05	Eosinophil adhesion and transendothelial migration in asthma	4.855E-04

The activation of AhR signaling pathways in the BALB/c 3T3 CTA model was not unexpected, as previously reported (Colacci et al., 2023). The canonical AhR pathway plays a role in both bioactivation and detoxification, potentially leading to or preventing oncotransformation *in vitro* (Mascolo et al., 2018; Pillo et al., 2022). Even in the absence of a recognizable formation of malignant foci, AhR is activated, indicating the modulation of several pathways associated with various potential adverse outcomes resulting from sustained inflammation. The upregulation of Cyp1A1 observed after 24 h of exposure and Cyp1B1 at 72 h confirmed the activation of the AhR canonical pathway.

The upregulation of Cyp2C enzymes, specifically Cyp2C9 and Cyp2C19, which are involved in human DEHP metabolism, confirmed the activation of PPARα. Indeed, CYP epoxygenases, including CYP2C and CYP2J, are affected by PPARα ligands (Cizkova et al., 2012). A fascinating notion is that Cyp2C enzymes are key molecules in the defensive response of embryonic and tumor cells, a phenomenon that translates into the mechanisms of multidrug resistance (MDR) in human pathophysiology. Upregulation of multidrug resistance protein 3 (MRP3), which is responsible for the transport of glucuronide conjugates and bile salts from the cell, can also confer resistance to several anticancer drugs (Aleo et al., 2017), further confirming that a series of key molecules in the cellular response to DEHP exposure move in unison in a string of genes correlated with the PXR pathway involved in the regulation of xenobiotic metabolism. Certain ligands or activators of PPARs affect the expression or activity of PXR and *vice versa*. The cooperative response observed when both RXR and partner receptor ligands are present highlights the regulatory interplay between permissive receptor partners, such as PPARs, PXR, and CAR (Evans and Mangelsdorf, 2014).

The high upregulation of Cyp2C8 highlights the interesting crosstalk between PPAR and AhR in our model. DEHP induces Cyp2C8 expression through the AhR genomic pathway, which is typically independent of ligand binding, and can interact with other transcription factors (Hsieh et al., 2022). The induction of Cyp2C8 increases epithelial-mesenchymal transition (EMT) sustained by the AhR/ERK signaling pathway (Hsieh et al., 2022). EMT plays a role in various biological processes under normal conditions such as embryogenesis and wound healing in adults (Colacci et al., 2023). However, they also contribute to the development of tissue fibrosis and cancer. In human cancers, EMT is considered a pivotal stage at the tissue level, signifying the progression of dysplasia and the acquisition of invasive characteristics (Colacci et al., 2023). It has previously been reported that CTAs, especially the BALB/c 3T3 model, offer the possibility of identifying critical steps related to EMT, a process that starts with cytoskeleton modifications as an adaptive response to chemical exposure and proceeds according to chemical concentration and exposure duration to extensive morphological changes, sustaining the acquisition of fully malignant characteristics (Colacci et al., 2023).

Based on our findings, we can infer that the molecular initiating event in our model involves the binding of DEHP to PPARα, which triggers a cascade of molecular events supporting DEHP metabolism and the AhR-mediated immune response. These pivotal molecular events are detectable only after 24 h of exposure, indicating that this timeframe allows for the early detection of chemical responses and the identification of molecular initiating events in *in vitro* oncogenesis.

At the 24 h mark, we also observed the activation of Ah-dependent detoxifying enzymes, specifically UDP-glucuronosyltransferase (phase 2 metabolic enzymes), Ugt1a1 and Ugt1a6. These enzymes facilitate glucuronidation, enabling the covalent attachment of glucuronic acid derived from the cofactor UDP-glucuronic acid to substrates containing appropriate acceptor functional groups. The upregulation of these enzymes confirms that DEHP metabolites are actively detoxified via glucuronidation, which is the primary route for the elimination of DEHP metabolites in humans.

Previous studies have indicated that the activation of UDP-glucuronosyltransferase plays a crucial role in detoxifying carcinogens in CTAs (Mascolo et al., 2018), and this serves as a unique signature of BALB/c 3T3 CTA (Colacci et al., 2023). Therefore, we can deduce that the negative results observed in the BALB/c 3T3 CTA compared with the SHE model can be attributed to the active detoxification of DEHP metabolites driven by a robust AhR-mediated response characteristic of the BALB/c 3T3 model.

BALB/c 3T3 cells, specifically the A31-1-1 clone, exhibit notable metabolic competence encompassing both phase-1 and phase-2 enzymes (Mascolo et al., 2018). This clone was deliberately selected for its superior metabolic capabilities compared with the A31-1-1 clone (Colacci et al., 2011; Colacci et al., 2023). Although initially believed to be derived from the A31 clone, subsequent characterization has revealed that it originated from a distinct mouse strain (Colacci et al., 2023). This discrepancy in lineage sheds light on the observed variations between the two clones and potentially explains the inconsistencies among studies that have utilized different clones. Moreover, these findings underscore the importance of considering the genetic background and metabolic characteristics of cell lines when interpreting toxicity data and highlight the need for continued research to elucidate the mechanistic basis of these differences.

After 72 h, all the genes associated with the AhR canonical pathway, including Cyp1A1, Cyp1B1, and UDP-glucuronosyltransferases, remained modulated, whereas the expression of genes linked to PPARα was negligible. Notably, the gene profiles associated with PPARγ were discernible at this juncture, which was surprising (Table 3).

## 5 Conclusion

The primary goal of this study was to evaluate the carcinogenic potential of DEHP using the A31-1-1 BALB/c-3T3 cell line in a standard CTA according to the ECVAM DB-ALM protocol No. 137 (Sasaki et al., 2012a; Corvi et al., 2012; Tanaka et al., 2012). Our investigation extended beyond CTA by incorporating a transcriptomic analysis to explore molecular responses. In this study, we examined the effects of DEHP exposure on various toxicological pathways. The results revealed significant modulation of several pathways associated with tissue-specific functions related to systemic metabolic and basal cellular signaling with pleiotropic outcomes. Among these signaling pathways, modulation of cell-regulating signaling pathways, such as Notch, Wnt, and TGF-β, can be highlighted. More specific modulation of such genes and pathways with double functions in metabolism and neurophysiology underlies a well-known crosstalk that may be crucial in the mechanism of action of DEHP. It is intriguing to note that such tissue-specific molecular signaling, which is known to be perturbed by DEHP, was scored in this enrichment analysis using mouse embryonic fibroblasts. Fibroblasts play a crucial role in tumor progression through their interactions within the tumor microenvironment. Once viewed solely as supportive cells that provide structural integrity, fibroblasts are now recognized as active participants in malignancy. Fibroblasts undergo activation to acquire the cancer-associated fibroblast (CAFs) phenotype. These CAFs secrete growth factors, cytokines, and extracellular matrix proteins that contribute to tumor growth, angiogenesis, invasion, and metastasis. The tumor microenvironment, characterized by dynamic and reciprocal interactions between cancer cells and the surrounding stromal cells, including fibroblasts, has emerged as a hallmark of cancer (Casey et al., 2015; Goodson et al., 2015). The ability of BALB/c 3T3 CTA to recapitulate key aspects of tumor progression, including the involvement of fibroblasts and key molecular events related to the tumor microenvironment, has been previously reported (Colacci et al., 2023) further emphasizing the relevance of CTA in delineating the multifaceted mechanisms underlying carcinogenesis.

In this study, the mode of action of DEHP related to the disruption of energy homeostasis, which has been previously described in mice, was similarly observed in the molecular toxicity data of 3T3 cells. The influence of PPARα molecular signaling on the modulated pathway was not statistically significant, despite the presence of several gene modulations associated with its signaling process. However, the upregulation of Cyp2C enzymes, integral to DEHP metabolism, directly regulated by PPARs, either independently or via crosstalk with AhR, strongly indicates that PPARα activation serves as the initiating event in our model. Moreover, persistent activation of the AhR canonical pathway throughout the exposure of cells to DEHP ensures sustained upregulation of detoxifying enzymes, thereby mitigating potential adverse effects induced by the chemical and preventing cell transformation.

**FIGURE 5:**
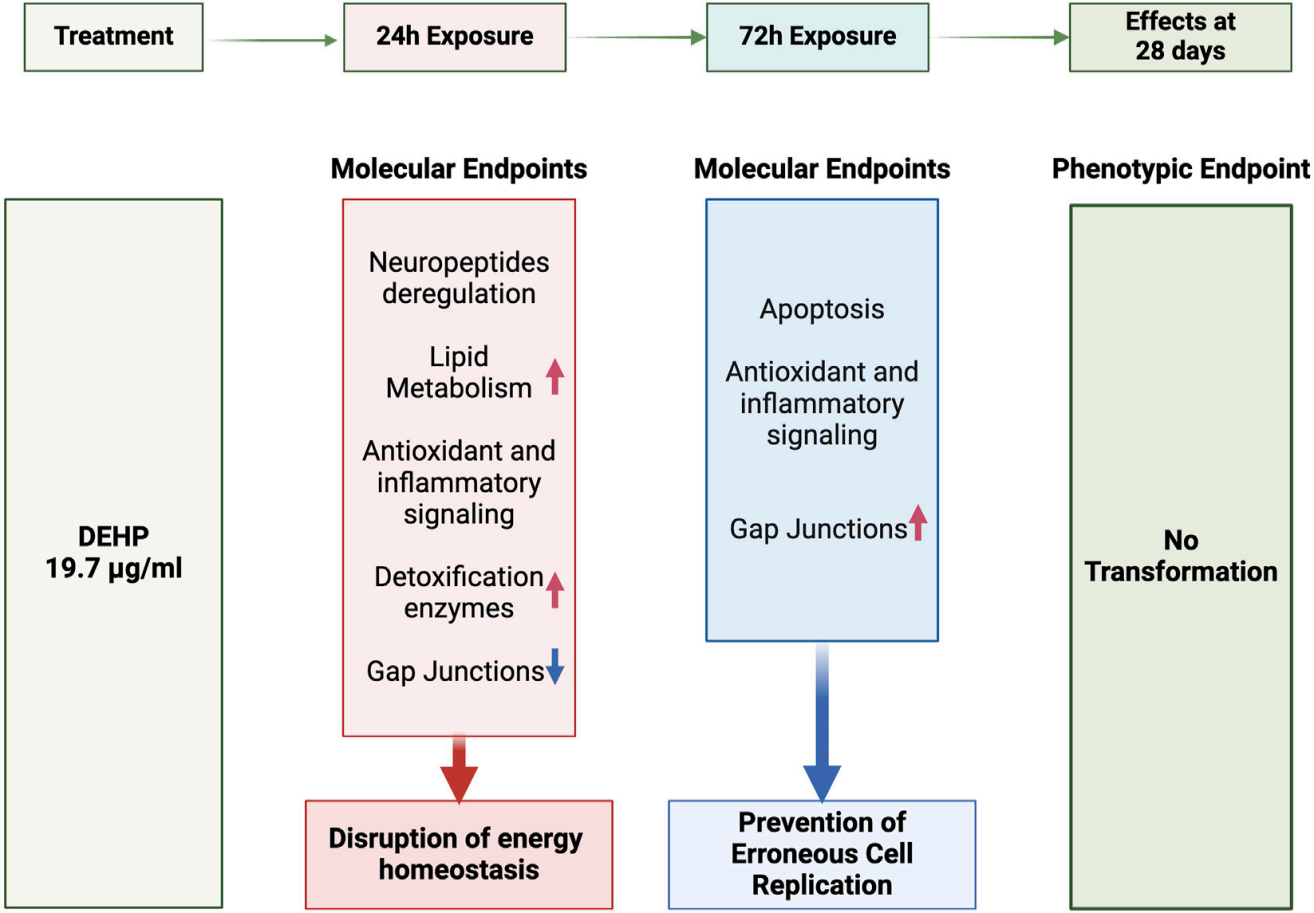
Temporal dynamics of key molecular endpoints during chemical treatment.

While the negative CTA result indicated that DEHP did not induce malignant transformation in cultured cells under specific experimental conditions, transcriptomic analysis provided deeper insights into the molecular responses elicited by DEHP. Indeed, transcriptomic data can offer a critical context for interpreting CTA results, aiding in the identification of the underlying molecular pathways associated with carcinogenicity. As previously documented (Colacci et al., 2023), the modulation of gene pathways supporting cell proliferation is anticipated to lead to oncotransformation *in vitro* (Colacci et al., 2023). Sustained cell proliferation can be regarded as a necessary hallmark, albeit not sufficient, for progression towards malignancy in CTA models as well as *in vivo* cancer processes (Colacci et al., 2023). The absence of clear signals related to sustained proliferation, particularly those supporting cytoskeleton remodeling-related EMT at 72 h, suggests interruption of the process leading to oncotransformation through the induction of apoptosis to prevent the replication of faulty cells (Figure 5).

According to our data, up to 24 h of exposure, the initiating event signals were still visible and prevailed in the cytotoxic and apoptotic signaling, which manifested at 72 h. This extended analysis aimed to delve deeper into the molecular mechanisms of action and their temporal evolution, thereby contributing to a more nuanced assessment of the toxicological implications of DEHP.

Therefore, our findings underscore the effectiveness of an integrated approach combining CTA with transcriptomics. This integration not only aids in interpreting results when CTA is employed as a standalone assay but also enhances the sensitivity and specificity of the test. In the context of a battery of tests such as the IATA, the likelihood of encountering false negatives (or false positives) is mitigated by the inclusion of multiple endpoints from diverse assays. Moreover, although CTA may serve as a component of the battery, integration of transcriptomic analysis can further enhance the predictive power of IATA.

In conclusion, the results of our study demonstrated that the BALB/c 3T3 A31-1-1 cell line does not exhibit a transformative effect in response to DEHP exposure. Nevertheless, our data revealed a nuanced molecular response to DEHP after 24 h of exposure, shedding light on the mechanisms underlying the metabolic activation and detoxification in our model. This underscores the potential contribution of AhR-mediated pathways to negative results in BALB/c 3T3 CTA. The identification of relevant metabolic pathways associated with human DEHP exposure further provides compelling evidence supporting the predictive capabilities of CTA models in assessing chemical toxicity in humans, thus offering promising avenues to reduce reliance on animal testing for toxicity assessments (Huang et al., 2017; Hansen and Piorczynski, 2019).

## Data Availability

The datasets presented in the study are deposited in the repository Array Express—Genomic Collection (https://www.ebi.ac.uk/biostudies/arrayexpress), accession number E-MTAB-13716.
