# Supplementary figures and images for: An insight into carcinogenic activity and molecular mechanisms of Bis(2-ethylhexyl) phthalate
Source: Front Toxicol. 2024 Jul 23;6:1389160. doi: 10.3389/ftox.2024.1389160 (PMC11300235; doi:10.3389/ftox.2024.1389160)

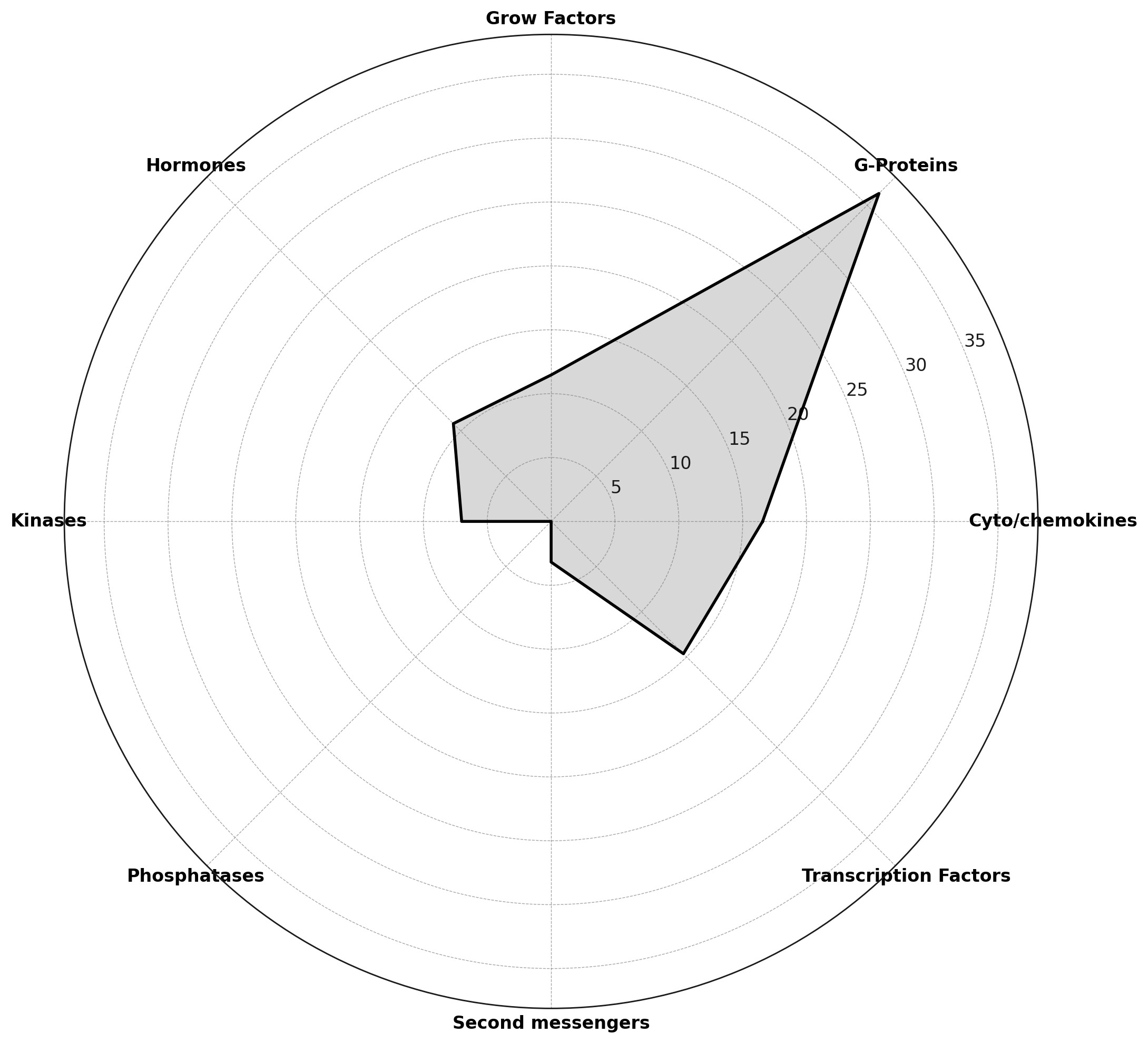

Supplement: Supplementary file 1 [file Image3.jpeg]

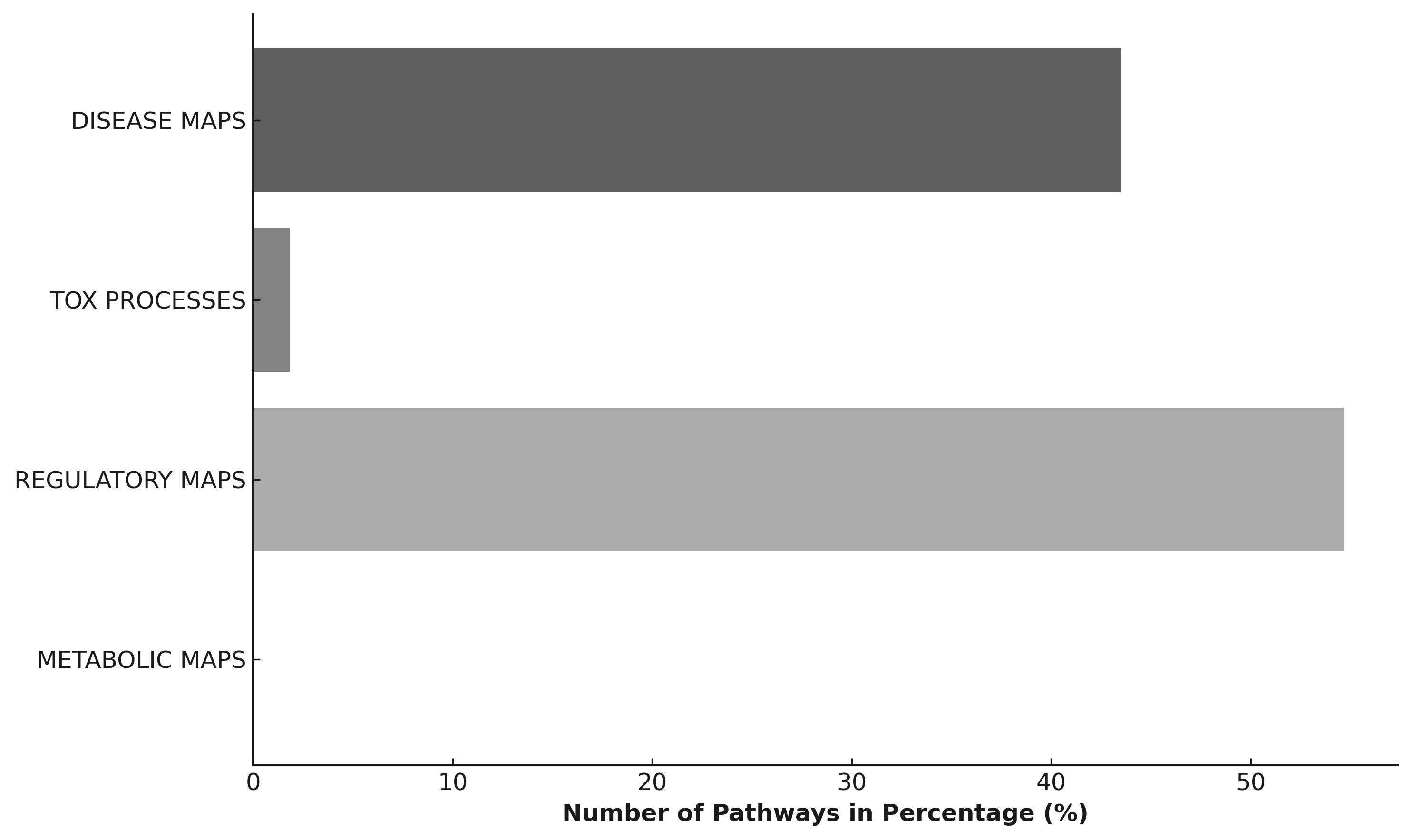

Supplement: Supplementary file 3 [file Image1.jpeg]

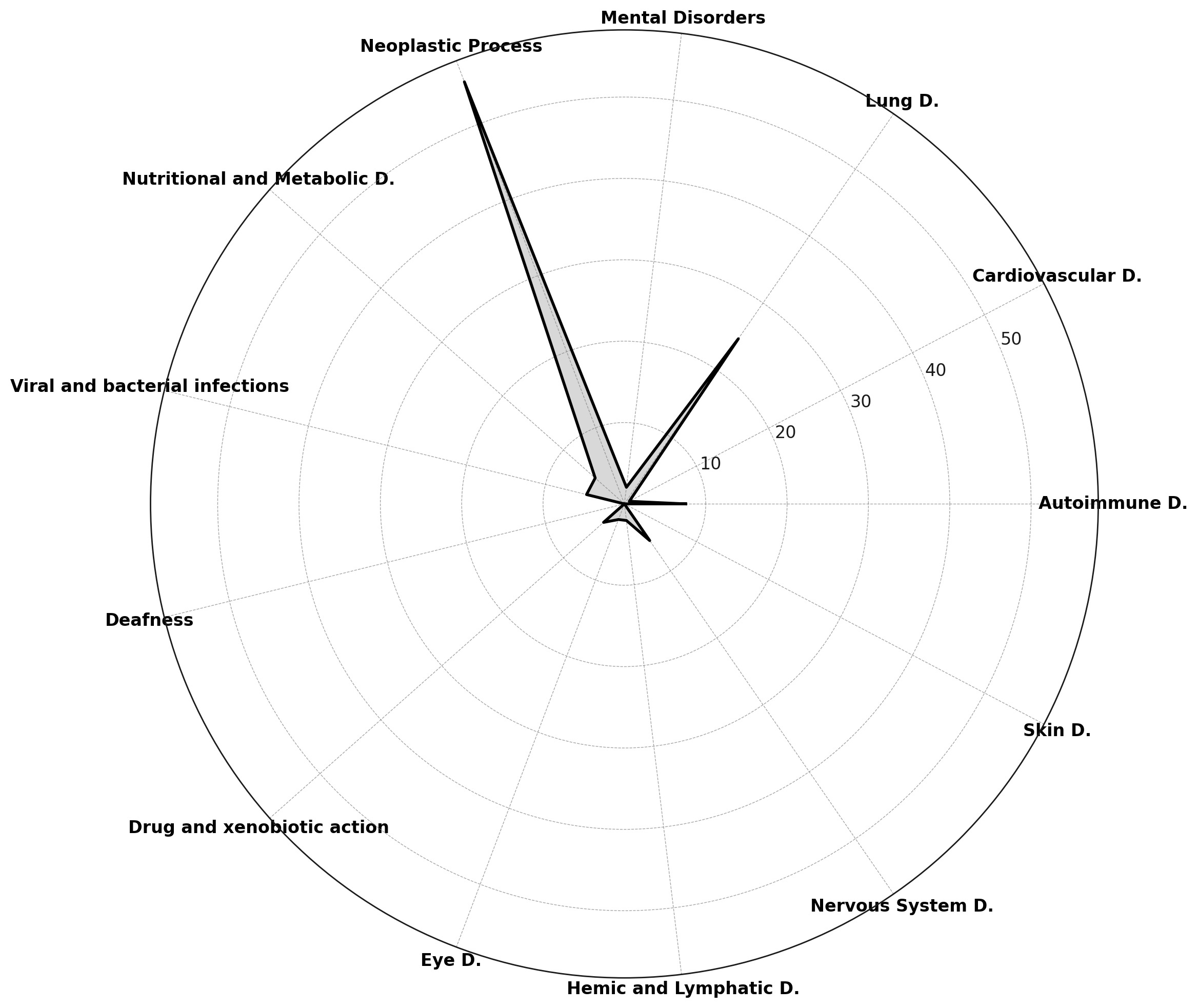

Supplement: Supplementary file 4 [file Image4.jpeg]

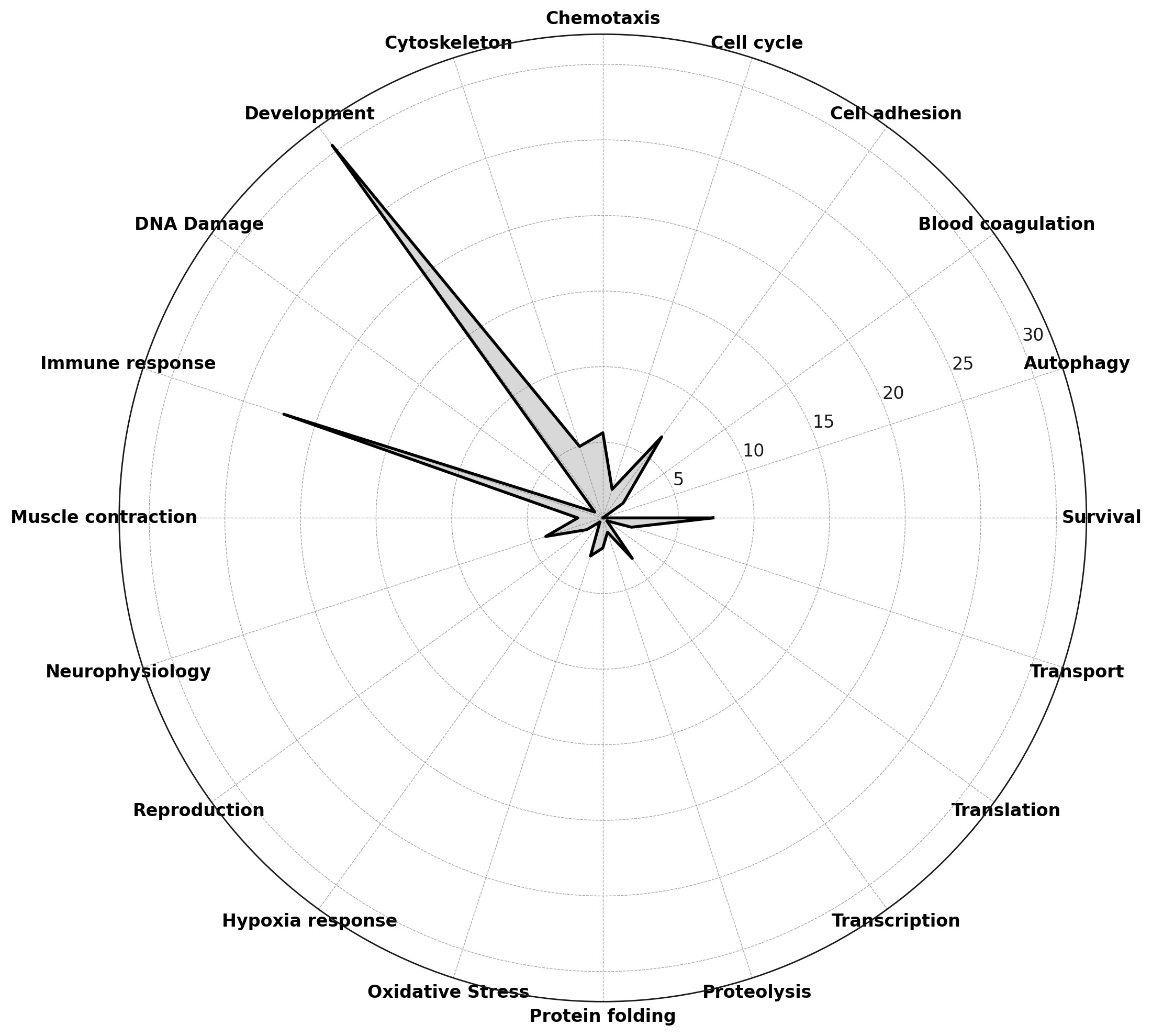

Supplement: Supplementary file 5 [file Image2.jpeg]
