# Supplementary material for: An insight into carcinogenic activity and molecular mechanisms of Bis(2-ethylhexyl) phthalate
Source: Front Toxicol. 2024 Jul 23;6:1389160. doi: 10.3389/ftox.2024.1389160 (PMC11300235; doi:10.3389/ftox.2024.1389160)
Supplement: Supplementary file 2 [file Table1.docx]

Supplementary Material

**An insight into carcinogenic activity and molecular mechanisms of Bis(2-ethylhexyl) phthalate**

**Gelsomina Pillo^1,2^, Federico Aldrovandi ^2,3^, Ada Mescoli ^2^, Giangabriele Maffei ^2,3^, Maria Grazia Mascolo ^1^, Monica Vaccari ^1^, Annamaria Colacci ^1,4^**

**^1^ Agency for Prevention, Environment and Energy (Arpae), Emilia-Romagna, Italy**

**^2^ Department of Medical and Surgical Sciences, DIMEC, Alma Mater Studiorum, University of Bologna**

**^3^Alma Mater Institute on Healthy Planet, University of Bologna, Italy**

**^4^Department of  Biological, Geological and Environmental Sciences, University of Bologna, Italy**

*** Correspondence:** Corresponding Author: [**annamaria.colacci@unibo.it**](mailto:annamaria.colacci@unibo.it)

**Table S1.** Literature Review of Cell Transformation Assay (CTA) Data Post DEHP Treatment, Including Relevant Protocol Information.

| **Study** | **Cellular model and Vehicle** | **Concentrations (µg/mL)** | **RCE %** | | **Foci (PT)** | **Method** | **Conclusions** | **Note** |
| --- | --- | --- | --- | --- | --- | --- | --- | --- |
|  |  |  | **S. A** | **CC. A** |  |  |  |  |
| Matthews et al., 1985 | Balb/c-3T3  Vehicle: Not reported | 0, 0.9, 3.5, 7.0, 12.0, 21.0   (-RLC^1^)  0, 19.6, 98.00, 9.00  (+ RLC^1^) | - | - | - | CTA  With and without metabolic activation details  Not reported | Negative | Results found in EUR 23384 EN_ 2008 |
| Matthews et al., 1993 | BALB/c-3T3 A31-1 13    Vehicle: F68 pluronic | 147 | 28.9 | 94.4 | 7 (18) | CTA  Chemial-induced transformation was detected in 18-20 vessels/dose seeded with 3.2 x 10^4^ cells/vessel. Treatment time: 48 hrs | Negative   Non-cytotoxic | Tests were repeated twice.  Solubility limit in medium reported: 12 µl/mL |
|  |  | 184 | 47.40 | 87.00 | 4 (18) |  |  |  |
|  |  | 29 | 20.9 | 92.3 | 2 (18) |  |  |  |
|  |  | 368 | 22.10 | 69.00 | 2 (18) |  |  |  |
|  |  | 590 | 6.81 | 70.3 | 1 (18) |  |  |  |
|  |  | 734 | 3.22 | 74.70 | 0 (18) |  |  |  |
|  |  | 11.75 x 10^3^ | 2.13 | 31.5 | 0 (18) |  |  |  |
|  |  | 14.72 x 10^3^ | 0.00 | 64.30 | 3 (17) |  |  |  |
|  |  | 0 | 100 | 100 | 20 (36) |  |  |  |
| **Study** | **Cellular model and Vehicle** | **Concentration (µg/mL)** | **Grow %** | | **Foci/well** | **Method** | **Conclusions** | **Note** |
|  |  |  |  |  |  |  |  |  |
| Kajiwara & Ajimib 2003 | BALB/c-3T3 A31-1-13  Vehicle: Acetone | 0 | 100 | | 0 | CTA  ITES-medium-improved^3^ BALB/c 3T3 cell transformation assay.  Treatment time: 24 hrs after seeding, for 4h and 24 hrs. Transformation assay: 5x10^5^ cells per 100-mm dish  Cells were replated after exposure using the ITES-medium and cultured for 20 days. | Negative | Solubility limit reported: 100 µg/mL |
|  |  | 15 | 67.2 | | 0 |  |  |  |
|  |  | 30 | 68.8 | | 0 |  |  |  |
|  |  | 60 | 71.7 | | 0.3 |  |  |  |
| **Study** | **Cellular model and Vehicle** | **Concentration (µg/mL)** | **RCE %** | | **Foci/well** | **Method** | **Conclusions** | **Note** |
| Nuodex, 1981f | BALB/c-3T3 A31    Vehicle: Not reported | 9.80, 98.50, 985 | - | | - | CTA details  Not reported  (-S9)^2^ | Positive | Results found in EUR 23384 EN_ 2008. Study performed under GLP |
|  |  | 98, 295.5, 980 | - | | - | CTA details  Not reported   (+S9)^2^ | Negative |  |
| Nuodex, 1981c | BALB/c-3T3  I13-C14    Vehicle: Not reported | 0.50, 4.98, 12.5, 24.9, 49.8 | - | | - | CTA details  Not reported | Negative | Results found in EUR 23384 EN_ 2008. Study performed under GLP |
| Nuodex, 1891d | BALB/c-3T3 I13-C14  Vehicle: Not reported | 1.64 – 32.8 | - | | - | CTA details  Not reported | Negative | Results found in EUR 23384 EN_ 2008. Study performed under GLP |
| Astill et al.,1986 | BALB/c-3T3  Vehicle: Not reported | 0.86 – 20.6 | - | | - | CTA details  Not reported | Negative | No solubility problem described |
| **Study** | **Cellular model and Vehicle** | **Concentration (µg/mL)** | **Viability %** | | **Foci/well** | **Method** | **Conclusions** | **Note** |
| Hwang et al., 2020 | Bhas 42    Vehicle: DMSO 0.1% | 0 | 100 | | 11.0 ± 1.79 | CTA (Promotion test)  Sakay et al., 2011 Protocol | Positive | No solubility problems described |
|  |  | 2.5 | 93 | | 13.8 ± 2.48 |  |  |  |
|  |  | 5 | 92 | | 19.5 ± 2.17** |  |  |  |
|  |  | 10 | 91 | | 20.5 ± 4.76** |  |  |  |
|  |  | 25 | 95 | | 18.5 ± 3.73** |  |  |  |
|  |  | 50 | 93 | | 18.5 ± 1.64** |  |  |  |
|  |  | 100 | 90 | | 13.0 ± 3.52 |  |  |  |
| **Study** | **Cellular model and Vehicle** | **Concentration (µg/mL)** | **Citotoxicity** | | **Foci /well** | **Method** | **Conclusions** | **Note** |
|  |  |  | **Plating Eff.** | **Relative P.E.** |  |  |  |  |
| Pant et al., 2010 | SHE  Vehicle: DMSO 0.2% | 0 | 31.4 | / | 5 | CTA  Treatment time:  7 day | Positive | The 100 µg/mL concentration formed precipitate in the treatment media |
|  |  | 2.5 | 34.6 | 110.2 | 13 |  |  |  |
|  |  | 5 | 33.3 | 106.2 | 14* |  |  |  |
|  |  | 10 | 34.4 | 109.6 | 18* |  |  |  |
|  |  | 25 | 21.8 | 69.6 | 12* |  |  |  |
|  |  | 50 | 7.8 | 24.9 | - |  |  |  |
|  |  | 100 | 27.1 | 86.4 | 6 |  |  |  |
| **Study** | **Cellular model and Vehicle** | **Concentration (µg/mL)** | **Viability %** | | **Foci/well** | **Method** | **Conclusions** | **Note** |
| LeBoeuf, 1996 | SHE  Vehicle: DMSO 0.2% | 0.625, 1.25, 2.5, 5 | - | | - | CTA  Treatment time:  24 hrs  7 days | Negative  (24 hrs)  Positive  (7 days ) | Lack of dose-response relationship  No solubility problem described |
| Tsutsui et al., 1993 | SHE  Vehicle: DMSO | 0 | 100 | | 0 | CTA  (-RLM^4^)  Cells were treated for 48 hrs in T75 flask. After treatment cells were plated on 100 mm dishes and incubated for 7 days | Positive | DMSO final concentration not reported |
|  |  | 1.17 | 101 | | 6* |  |  |  |
|  |  | 3.90 | 107 | | 6* |  |  |  |
|  |  | 11.72 | 104 | | 4 |  |  |  |
|  |  | 39.06 | 62 | | 1 |  |  |  |
|  |  | 0 | 100 | | 0 | CTA  (+RLM^4^)  Cells were seeded and 100 mm dishes  Treatment times: 2hrs  Cells were then incubated for 4 hrs | Positive |  |
|  |  | 1.17 | 120 | | 1 |  |  |  |
|  |  | 3.90 | 95 | | 2 |  |  |  |
|  |  | 11.72 | 90 | | 13** |  |  |  |
|  |  | 39.06 | 87 | | 13** |  |  |  |
| Jones et al., 1988 | SHE  Vehicle: Medium or DMSO 0.2% | 13– 4000 | - | | - | CTA  Treatment time:  24 hrs after seeding for 7 days | Positive (concentrations > 1 mg/mL) | Interlaboratory Test:  3 Labs  No solubility problem described  Poor cytotoxic dose-response relationship |
| **Study** | **Cellular model and Vehicle** | **Concentration (µg/mL)** | **Viability %** | | **Foci** | **Method** | **Conclusions** | **Note** |
| Mikalsen et al., 1990 | SHE  Vehicle: DMSO 0.2% | 0, 9.76, 19.53, 29.29 | - | | 1.9% morphological transformation for the highest concentration | CTA  Treatment time:  24 hrs after seeding for 7 days | Positive  Non-cytotoxic | Solubility problem at higher concentrations |
| Sanchez et al., 1987 | C3H/10T1/2  Vehicle: DMSO 0.5% | 1.95 | 98 | |  | CTA (initiation/promotion)  (20,000 cells/60 mm dish)  Treatment time:  24 hrs after seeding for 24 hrs | Negative |  |
|  |  | 3.90 | 109 | |  |  |  |  |
|  |  | 19.53 | 83 | |  |  |  |  |
|  |  | 29.29 | 30 | |  |  |  |  |
|  |  | 39.05 | 1 | |  |  |  |  |
| Steele et al., 1989 | Primary rat tracheal epithelial cells  Vehicle: DMSO 0.2% | 37.5 | IC50 ~ 107 µg/mL | |  | CTA  (20,000 cells/60 mm dish)  Treatment time:  24 hrs after seeding for 24 hrs | Positive |  |

** p < 0.01 and * p < 0.05 as compared with vehicle control (different tests)

^1^ Metabolic supplementation: feeder-layer of X- irradiated Rat Liver Cells (RLC)

^2^  Metabolic supplementation: With (+) and without (-) the use of S9 subcellular enzyme fractions

^3^ T medium or DME.F-12 medium supplemented with a mixture of insulin, transferrin, ethanolamine and sodium selenite (ITES) and 2% fetal bovine serum (FBS) (ITES-medium) is used during the period of expression of transformed foci.

^4^ Metabolic supplementation: With (+) and without (-) Rat liver microsome (RLM) fractions

**LEGEND:**

RCE%: Relative Clonal Efficiency

S. A: Standard Clonal Survival Assay

# CC. A: Co-culture Clonal Survival Assay

# CTA: Cell Transformation Assay

# Plating Eff.: Plating efficiency

# Relative P.E.: Relative Plating efficiency

IC50: half maximal inhibitory concentration

#

# 
